# Human and murine fibroblast single-cell transcriptomics reveals fibroblast clusters are differentially affected by ageing and serum cholesterol

**DOI:** 10.1093/cvr/cvad016

**Published:** 2023-01-31

**Authors:** Kim van Kuijk, Ian R McCracken, Renée J H A Tillie, Sebastiaan E J Asselberghs, Dlzar A Kheder, Stan Muitjens, Han Jin, Richard S Taylor, Ruud Wichers Schreur, Christoph Kuppe, Ross Dobie, Prakesh Ramachandran, Marion J Gijbels, Lieve Temmerman, Phoebe M Kirkwoord, Joris Luyten, Yanming Li, Heidi Noels, Pieter Goossens, John R Wilson-Kanamori, Leon J Schurgers, Ying H Shen, Barend M E Mees, Erik A L Biessen, Neil C Henderson, Rafael Kramann, Andrew H Baker, Judith C Sluimer

**Affiliations:** Cardiovascular Research Institute Maastricht (CARIM), Maastricht University Medical Center, PO Box 5800, 6202 AZ Maastricht, The Netherlands; Institute of Experimental Medicine and Systems Biology, Faculty of Medicine, RWTH Aachen University, Aachen, Germany; BHF Centre for Cardiovascular Sciences (CVS), Queen's Medical Research Institute, University of Edinburgh, 47 Little France Crescent, Edinburgh EH16 4TJ, UK; Cardiovascular Research Institute Maastricht (CARIM), Maastricht University Medical Center, PO Box 5800, 6202 AZ Maastricht, The Netherlands; Cardiovascular Research Institute Maastricht (CARIM), Maastricht University Medical Center, PO Box 5800, 6202 AZ Maastricht, The Netherlands; Department of Vascular Surgery, Maastricht University Medical Center, Maastricht, The Netherlands; Cardiovascular Research Institute Maastricht (CARIM), Maastricht University Medical Center, PO Box 5800, 6202 AZ Maastricht, The Netherlands; Cardiovascular Research Institute Maastricht (CARIM), Maastricht University Medical Center, PO Box 5800, 6202 AZ Maastricht, The Netherlands; Cardiovascular Research Institute Maastricht (CARIM), Maastricht University Medical Center, PO Box 5800, 6202 AZ Maastricht, The Netherlands; BHF Centre for Cardiovascular Sciences (CVS), Queen's Medical Research Institute, University of Edinburgh, 47 Little France Crescent, Edinburgh EH16 4TJ, UK; Cardiovascular Research Institute Maastricht (CARIM), Maastricht University Medical Center, PO Box 5800, 6202 AZ Maastricht, The Netherlands; Institute of Experimental Medicine and Systems Biology, Faculty of Medicine, RWTH Aachen University, Aachen, Germany; Division of Nephrology and Clinical Immunology, Faculty of Medicine, RWTH Aachen University, Aachen, Germany; Centre for Inflammation Research, University of Edinburgh, Edinburgh, UK; Department of Medical Biochemistry, Experimental Vascular Biology, Amsterdam UMC, Amsterdam, The Netherlands; Cardiovascular Research Institute Maastricht (CARIM), Maastricht University Medical Center, PO Box 5800, 6202 AZ Maastricht, The Netherlands; Department of Medical Biochemistry, Experimental Vascular Biology, Amsterdam UMC, Amsterdam, The Netherlands; GROW, School for Oncology and Development Biology, Maastricht University, Maastricht, The Netherlands; Cardiovascular Research Institute Maastricht (CARIM), Maastricht University Medical Center, PO Box 5800, 6202 AZ Maastricht, The Netherlands; Centre for Inflammation Research, University of Edinburgh, Edinburgh, UK; Cardiovascular Research Institute Maastricht (CARIM), Maastricht University Medical Center, PO Box 5800, 6202 AZ Maastricht, The Netherlands; Department of Vascular Surgery, Maastricht University Medical Center, Maastricht, The Netherlands; Division of Cardiothoracic Surgery, Baylor College of Medicine, Houston, TX, USA; Department of Cardiovascular Surgery, Texas Heart Institute, Houston, TX, USA; Cardiovascular Research Institute Maastricht (CARIM), Maastricht University Medical Center, PO Box 5800, 6202 AZ Maastricht, The Netherlands; Cardiovascular Research Institute Maastricht (CARIM), Maastricht University Medical Center, PO Box 5800, 6202 AZ Maastricht, The Netherlands; Division of Nephrology and Clinical Immunology, Faculty of Medicine, RWTH Aachen University, Aachen, Germany; Cardiovascular Research Institute Maastricht (CARIM), Maastricht University Medical Center, PO Box 5800, 6202 AZ Maastricht, The Netherlands; Institute of Experimental Medicine and Systems Biology, Faculty of Medicine, RWTH Aachen University, Aachen, Germany; Division of Cardiothoracic Surgery, Baylor College of Medicine, Houston, TX, USA; Department of Cardiovascular Surgery, Texas Heart Institute, Houston, TX, USA; Cardiovascular Research Institute Maastricht (CARIM), Maastricht University Medical Center, PO Box 5800, 6202 AZ Maastricht, The Netherlands; Department of Vascular Surgery, Maastricht University Medical Center, Maastricht, The Netherlands; Cardiovascular Research Institute Maastricht (CARIM), Maastricht University Medical Center, PO Box 5800, 6202 AZ Maastricht, The Netherlands; Institute for Molecular Cardiovascular Research, RWTH Aachen University, Aachen, Germany; Division of Nephrology and Clinical Immunology, Faculty of Medicine, RWTH Aachen University, Aachen, Germany; MRC Human Genetics Unit, Institute of Genetics and Cancer, University of Edinburgh, Edinburgh, UK; Institute of Experimental Medicine and Systems Biology, Faculty of Medicine, RWTH Aachen University, Aachen, Germany; Department of Vascular Surgery, Maastricht University Medical Center, Maastricht, The Netherlands; Cardiovascular Research Institute Maastricht (CARIM), Maastricht University Medical Center, PO Box 5800, 6202 AZ Maastricht, The Netherlands; BHF Centre for Cardiovascular Sciences (CVS), Queen's Medical Research Institute, University of Edinburgh, 47 Little France Crescent, Edinburgh EH16 4TJ, UK; Cardiovascular Research Institute Maastricht (CARIM), Maastricht University Medical Center, PO Box 5800, 6202 AZ Maastricht, The Netherlands; BHF Centre for Cardiovascular Sciences (CVS), Queen's Medical Research Institute, University of Edinburgh, 47 Little France Crescent, Edinburgh EH16 4TJ, UK

**Keywords:** Adventitia, Fibroblasts, Heterogeneity, Atherosclerosis, Single-cell RNA-seq

## Abstract

**Aims:**

Specific fibroblast markers and in-depth heterogeneity analysis are currently lacking, hindering functional studies in cardiovascular diseases (CVDs). Here, we established cell-type markers and heterogeneity in murine and human arteries and studied the adventitial fibroblast response to CVD and its risk factors hypercholesterolaemia and ageing.

**Methods and results:**

Murine aorta single-cell RNA-sequencing analysis of adventitial mesenchymal cells identified fibroblast-specific markers. Immunohistochemistry and flow cytometry validated platelet-derived growth factor receptor alpha (PDGFRA) and dipeptidase 1 (DPEP1) across human and murine aorta, carotid, and femoral arteries, whereas traditional markers such as the cluster of differentiation (CD)90 and vimentin also marked transgelin+ vascular smooth muscle cells. Next, pseudotime analysis showed multiple fibroblast clusters differentiating along trajectories. Three trajectories, marked by CD55 (*Cd55*+), Cxcl chemokine 14 (*Cxcl14*+), and lysyl oxidase (*Lox+*), were reproduced in an independent RNA-seq dataset. Gene ontology (GO) analysis showed divergent functional profiles of the three trajectories, related to vascular development, antigen presentation, and/or collagen fibril organization, respectively. Trajectory-specific genes included significantly more genes with known genome-wide associations (GWAS) to CVD than expected by chance, implying a role in CVD. Indeed, differential regulation of fibroblast clusters by CVD risk factors was shown in the adventitia of aged C57BL/6J mice, and mildly hypercholesterolaemic LDLR KO mice on chow by flow cytometry. The expansion of collagen-related CXCL14+ and LOX+ fibroblasts in aged and hypercholesterolaemic aortic adventitia, respectively, coincided with increased adventitial collagen. Immunohistochemistry, bulk, and single-cell transcriptomics of human carotid and aorta specimens emphasized translational value as CD55+, CXCL14+ and LOX+ fibroblasts were observed in healthy and atherosclerotic specimens. Also, trajectory-specific gene sets are differentially correlated with human atherosclerotic plaque traits.

**Conclusion:**

We provide two adventitial fibroblast-specific markers, PDGFRA and DPEP1, and demonstrate fibroblast heterogeneity in health and CVD in humans and mice. Biological relevance is evident from the regulation of fibroblast clusters by age and hypercholesterolaemia *in vivo*, associations with human atherosclerotic plaque traits, and enrichment of genes with a GWAS for CVD.

## Graphical Abstract

**Graphical Abstract:**
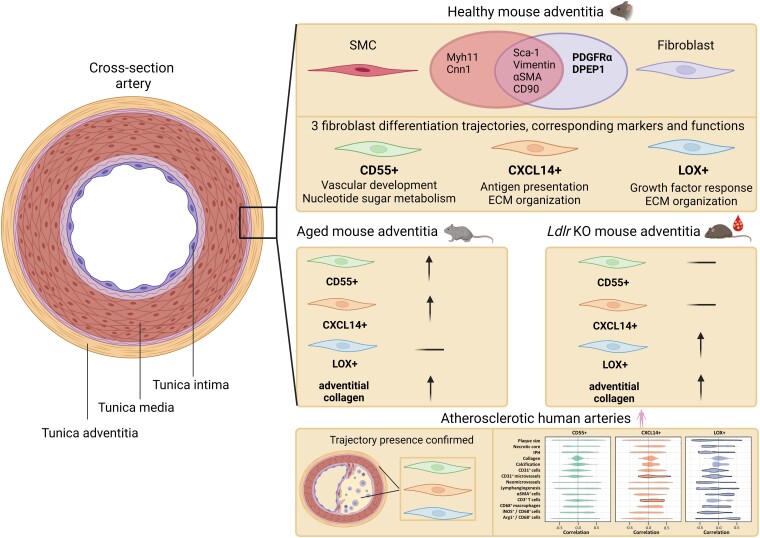

## 1. Introduction

Cellular heterogeneity and plasticity are two fundamental concepts that are beginning to define both the healthy and diseased vasculature.^1^ This challenges the traditional approach to understanding previously distinct cellular compartments in the blood vessel wall, and the identities of cells that infiltrate the vessel wall in disease.^2^ One cell type in particular, known for its high plasticity and heterogeneity in numerous organs, is the fibroblast.^3-5^ Fibroblasts mostly reside in the adventitial layer of the arterial wall, accompanied by other mesenchymal cells [e.g. pericytes and smooth muscle cells (SMCs)], immune cells, and connective tissue.^6^ Mainly fibroblasts express the stem cell marker Sca-1/Ly6a, underpinning the potential of these cells to be reprogrammed into a diverse cell repertoire, supporting extensive plasticity.^7,8^ Their functional role in fibrosis, inflammation, and angiogenesis in other organs^9,10^ makes these cells an attractive candidate for therapeutic intervention in arterial pathologies, such as atherosclerosis and vascular ageing. However, presumably also due to this plasticity, markers specifically distinguishing fibroblasts at the mRNA and protein level from other vascular cells have been very difficult to define. For example, traditional markers such as collagen 1 alpha 1 (*Col1α1*), collagen 1 alpha 2 (*Col1α2*), fibroblast activation protein (*Fap*), and fibroblast-specific protein-1 (*Fsp-1*) lack the ability to distinguish between fibroblasts and other vascular cell types.^11^ In addition, other vascular mesenchymal cells exhibit phenotypes resembling that of fibroblasts upon vascular challenges.^12,13^ Nevertheless, these markers have been used to detect fibroblast-like cells, originating from SMCs, or endothelial cells in atherosclerosis.^13-15^ Thus, there is a need to resolve their fibroblast specificity to discern the impact or limitations of these studies. In addition, the role and regulation of potential fibroblast heterogeneity in vascular health and disease is not explored in sufficient detail but understanding disease-stimulating or -preventing phenotypes may impact therapeutic approaches.

Single-cell RNA-sequencing (scRNA-seq) and concomitant extensive validation could resolve the ambiguity of fibroblast identity markers and potential heterogeneity. Indeed, scRNA-seq has been key in identifying pan-fibroblast-specific markers across the microvasculature in several major organs compared with mural cells (MCs) (consisting of pericytes and SMCs).^16^ Yet, it remains to be defined which markers are specific for arterial adventitial fibroblasts compared with other arterial cells. Previous scRNA-seq analyses of healthy murine vasculature have described transcriptomics of all arterial wall cell types, including fibroblasts, in a so-called atlas approach.^17,18^ While both studies propose cell identity markers and indicate the presence of multiple fibroblast clusters, the data stem from a low number of fibroblasts, and results are not comprehensively validated on protein level. We hypothesize that a very detailed analysis of arterial fibroblasts would improve the definition of fibroblast identity markers and detailed insight into fibroblast heterogeneity.

In the current study, we, therefore, investigated the fibroblast transcriptional landscape using scRNA-seq of fibroblast-enriched fractions from healthy murine adventitia. Fibroblast heterogeneity and pseudotime differentiation trajectories were analysed in-depth by bioinformatic analyses, such as Potential of Heat-diffusion for Affinity-based Trajectory Embedding (PHATE). The identified fibroblast identity and cluster markers were validated extensively on RNA and protein level using bulk and single-cell sequencing, flow cytometry, and immunohistochemistry of murine and human healthy and atherosclerotic arteries. We provide support for regulation of fibroblast heterogeneity in cardiovascular disease (CVD), as cardiovascular (CV) risk factors differentially affected fibroblast cluster expansion in aged and hypercholesterolaemic mice *in vivo*, cluster gene signatures harboured a significant number of genes with a known genome-wide associations (GWAS) to CVD, and were associated with human atherosclerotic plaque traits. Together, this study provides a detailed fingerprint of arterial fibroblasts in health and CVD.

## 2. Methods

Full methods can be found in the online data supplement.

### 2.1 Mouse models

All mouse experiments were approved by the regulatory authority of the Maastricht University Medical Centre and performed in compliance with the Dutch governmental guidelines and Directive 2010/63/EU of the European Parliament on the protection of animals used for scientific purposes. C57BL/6J mice (male, *n* = 8 per pool, 3–4 pools, 8–12 weeks old) were used as healthy controls. Aged C57BL/6J mice (male, *n* = 5 per pool, 3–4 pools, 72 weeks old) were obtained from Charles River and used to study the effect of ageing. Male low-density lipoprotein (LDL) receptor-deficient knock-out (*Ldlr* KO) mice were fed chow (controls) or high-cholesterol diet (HCD, 0.25%, 824171, Tecnilab-BMI) for 16 weeks (*n* = 15 per pool for single-cell sequencing, *n* = 5 per pool, 3 pools for flow cytometry, 28–30 weeks old). *Ldlr* KO mice originated from Jaxx and were bred in Maastricht for <15 generations. Pdgfrα-CreERT2-Rosa26-tdTomato and Myh11-CreERT2 eYFP were intraperitoneally injected with Tamoxifen (200 mg/kg) for three consecutive days, to induce TdTomato expression. Mice were euthanized with an overdose of pentobarbital (100 mg/kg) injected intraperitoneally.

### 2.2 Flow cytometry and cell sorting

Adventitia of the thoracic aorta [ranging from the aortic root until the diaphragm (see Supplementary material online, *Video S1*)] was carefully microscopically dissociated from the underlying medial layer and collected in ice-cold phosphate buffered saline. Adventitial tissue of C57BL/6J or *Ldlr* KO mice was enzymatically digested for 15 min at 37°C using collagenase B (0.00284 g/mL), pronase (0.01 g/mL), and DNAse (0.1 mg/mL). Living DAPI−, ICAM2−, and CD45− cells were sorted in the case of 8-week-old C57BL/6J mice or DAPI− cells for *Ldlr* KO.

Adventitial cells isolated originating from either young C57BL/6J mice (8 weeks, male), aged C57BL/6J mice (72 weeks, male), and Ldlr KO mice on chow or HCD for 16 weeks were used for protein validation. After FC receptor blocking, cells were stained with the following antibodies: CD45, Cdh5/VE-cadherin, Transgelin (TGLN), platelet-derived growth factor alpha (PDGFRA), CD55, CXCL14, and lysyl oxidase (LOX), live/dead fixable cell stain. In the case of CXCL14, the antibody was labelled using a PE/Cy7 conjugation kit. For intracellular staining (Transgelin, CXCL14, and LOX), a fix & perm cell permeabilization kit was used. Data analysis was performed with BD FACS Diva software.

### 2.3 Immunohistochemical staining

Murine tissue was fixed in 1% paraformaldehyde overnight, paraffin-embedded, and serially sectioned (4 µm). For staining, only sections that had mature media (determined by elastin fibre presence) were used. Tissue sections were stained for the following proteins: SMOC2, PDGFRA, FBLN1, LUMICAN, CCL11, DPEP1, MAC3, CD55, CXCL14, LOX, COL1A1, and total collagen. Images were analysed either with Qupath (v0.2.0-m8) or Leica Qwin software.

### 2.4 Human sample analysis

Human tissue collection was part of the Maastricht Pathology Tissue Collection (MPTC) and further storage and use of the tissue was in line with the Dutch Code for Proper Secondary Use of Human Tissue and the local Medical Ethics Committee (protocol number 16-4-181). This code (https://elsi.health-ri.nl/sites/elsi/files/2022-01/Gedragscode_Gezondheidsonderzoek_2022.pdf) entails an opt-out arrangement and hence tissues were not used in the case of objection. The applicability of this code for this study was approved by the Maastricht University hospital (MUMC) local Medical Ethics Committees. Human studies conducted by Li *et al*.^19^ and Wirka *et al*.^13^ are approved by the Institutional Review Board at Baylor College of Medicine and Stanford University Institutional Review Board, respectively, and follow the guidelines of the Declaration of Helsinki. Written informed consent was provided by all participants or the organ donors’ legal representatives before enrolment. Carotid samples were collected either through autopsy (*n* = 10), carotid endarterectomy procedure (*n* = 63 from 43 patients), from the opposite side of the plaque (*n* = 10), or during aortic bypass surgery (*n* = 10). Library preparation, RNA extraction, data processing, normalization, and additional information concerning plaque traits have been described in great detail elsewhere.^20,21^ Human carotid and aorta single-cell sequencing data were retrieved from data repositories and analysed according to published methods.^13,19^

### 2.5 Murine single-cell sequencing

After cell count number and viability check with Trypan Blue (>85%), a total of ∼16.000 CD45−/ICAM2− cells of C57BL/6J mice (*n* = 8) and ∼15.000 cells of *Ldlr* KO mice (*n* = 13 for HCD group and *n* = 15 for chow group) were loaded on a chromium 10 × genomics controller (V2). Libraries were synthesized and sequenced using Illumina HiSeq4000. Cell and gene number per sample can be found in Supplementary material online, *Tables S1* and *S2*.

### 2.6 Single-cell sequencing analysis

Raw sequencing data were processed using CellRanger (v2.1.1 for C57BL/6J mice and v3.0.2 for *Ldlr* KO mice) and analysed using R and Seurat R package (v.2.3 for C57BL/6J mice and v3.2.3 for *Ldlr* KO mice), and G:profiler^22^ for GO analysis of biological processes. Pseudotime analysis was done with PHATE dimension reduction,^23^ RNA velocity,^24^ and Monocle (v.2.10.1).^25^ Full details of the analysis can be found in the online data supplement.

### 2.7 Enrichment analysis

DEGs from full trajectories (F1, F2, F3, F4, *n* = 216; F5, F6, F7, *n* = 235; F8, F9, *n* = 317) were intersected with (i) Genome wide association studies (GWAS) coronary artery disease (CAD)-associated genes and (ii) human aorta fibroblast DEGs originating from the study by Li *et al*.^19^ Hypergeometric testing was used to evaluate the statistical significance of the overlap between trajectory genes and CAD or fibroblast genes. Mouse genes were converted to human genes by biomaRt R package (v2.50.1).^26^

## 3. Results

### 3.1 ScRNA-seq yields a seven-marker signature differentially regulated in fibroblasts compared with other cells in murine healthy vasculature

**Figure 1:**
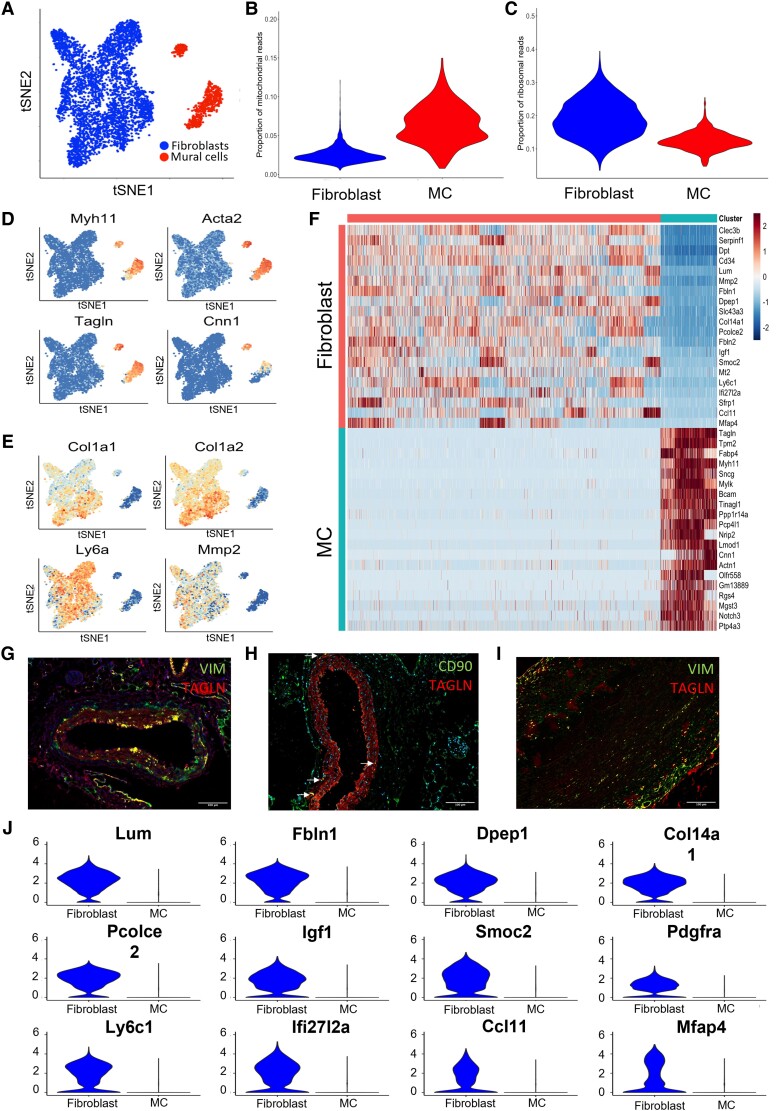
scRNA-seq reveals fibroblast transcriptional signature for healthy murine aortic adventitia. (*A*) T-distributed stochastic neighbour embedding (tSNE) plot of single-cell sequencing data derived from CD45−/ICAM2−/PDGFRβ+ adventitial cells from a pool of nine young C57Bl6 mice. (*B*) Mitochondrial signature of fibroblasts and MCs post-filtering; (*C*) ribosomal signature of fibroblasts and MC post-filtering; (*D*) expression of MC markers (*Myh11, Acta2, Tgln, Cnn1*); and (*E*) traditional fibroblast markers (*Col1α1, Col1α2, Ly6a, Mmp2)* projected on tSNE plot from *D* shows cell-type annotation. (*F*) Heatmap of differentially expressed genes (DEGs) in fibroblasts and MC. Immunohistochemical staining of SMC marker Tgln (red) with traditional fibroblast markers (green) in mice. (*G*) Vimentin (VIM), (*H*) CD90, and (*I*) human aorta (VIM) (*J*). Violin plots of 12 genes differentially expressed in fibroblasts compared with MC.

The adventitia of the thoracic aorta from eight healthy male C57Bl/6J mice was collected and pooled for isolation of DAPI−, CD45^−^, and intercellular adhesion molecule 2 (ICAM2)^−^ cells to exclude immune and endothelial cells and enrich for the viable, mesenchymal population prior to ScRNA analysis (see Supplementary material online, *Figure S1A–C* and *Video S1*). This approach allowed in-depth analysis of adventitial mesenchymal cells. In total, 5700 cells passed single-cell RNA quality control after the removal of low-quality cells (<1500 genes, >15% mitochondrial reads), and potential doublets (UMI count > 15 000) (Supplementary material online, *Tables S1* and *S2*, *Figure 1A–C*). Firstly, *in silico* selection of mesenchymal cells was done, based on *Pdgfrβ* expression (see Supplementary material online, *Figure S1A*). Subsequently, annotation of the identified clusters was based on previously published markers for MCs [Myosin heavy chain 11 (*Myh11*), Transgelin (*Tagln*), Alpha actin (*Acta2*), and calponin (*Cnn1*)] and fibroblasts [*Col1a1, Col1a2*, Matrix metalloproteinase 2 (*Mmp2*), and Stem cell antigen-1 (*Sca-1/Ly6a*)^16^]. These markers confirmed the presence of both fibroblasts and MCs in healthy mouse adventitia (*Figure 1D and E*). The absence of macrophage (*Cd68*), endothelial cell [platelet endothelial cell adhesion molecule-1 (*Pecam1*)], neuron [RNA binding protein, fox-1 homolog 3 (*Rbfox3*)], and adipocyte [Adiponectin (*Adipoq*)] markers confirmed the purity of our sorting strategy (see Supplementary material online, *Table S3*). Differential gene expression analysis comparing fibroblast and MC populations revealed distinct expression profiles for both cell types (*Figure 1F*). Subsequent GO enrichment analysis based on differentially expressed genes returned terms including ‘extracellular matrix’ and ‘contractile fibre’ corresponding to fibroblast and MC populations, respectively (see Supplementary material online, *Figure S1D* and *E*).

Notably, many of the commonly proposed fibroblast markers from the literature, including vimentin (VIM), matrix metalloproteinase-2 (MMP2), CD90, Sca-1, and FAP, were not able to fully differentiate between fibroblasts and MCs, as evidenced by RNA expression in pericytes and SMCs in three other single-cell RNA datasets (see Supplementary material online, *Figure S2A* and *B*). Despite RNA levels being higher in fibroblasts than MCs, protein co-expression with TAGLN+ SMCs was observed in healthy human and murine aorta (*Figure 1G–I*, Supplementary material online, *Figure S2C–E*). Thus, we next assessed genes differentially expressed between fibroblasts and MCs to create a fibroblast-specific transcriptional signature. Analysis of Differential expressed genes (DEG) analysis provided 12 markers preferentially expressed in adventitial fibroblasts (*Figure 1J*). Enrichment of seven of these markers [*Pdgfra,* Dipeptidase 1 (*Dpep1*), SPARC-related modular calcium binding 2 (*Smoc2*), Collagen 14 alpha 1 (*Col14a1*), Fibulin 1 (*Fbln1*), Lumican (*Lum*) and C-C Motif Chemokine Ligand 11 (*Ccl11*)] for mesenchymal fibroblasts remained after validation in two other available scRNA-seq datasets^18,27^ (see Supplementary material online, *Figure S1F* and *G*). Taken together, seven fibroblast markers (*Pdgfra, Lum, Smoc2, Col14a1, Fbln1, Dpep1, and Ccl11*) selected from our dataset were also expressed in fibroblasts and/or mesenchymal cells in two other datasets comprising healthy murine vasculature and a database including multiple murine organs.

**Figure 2:**
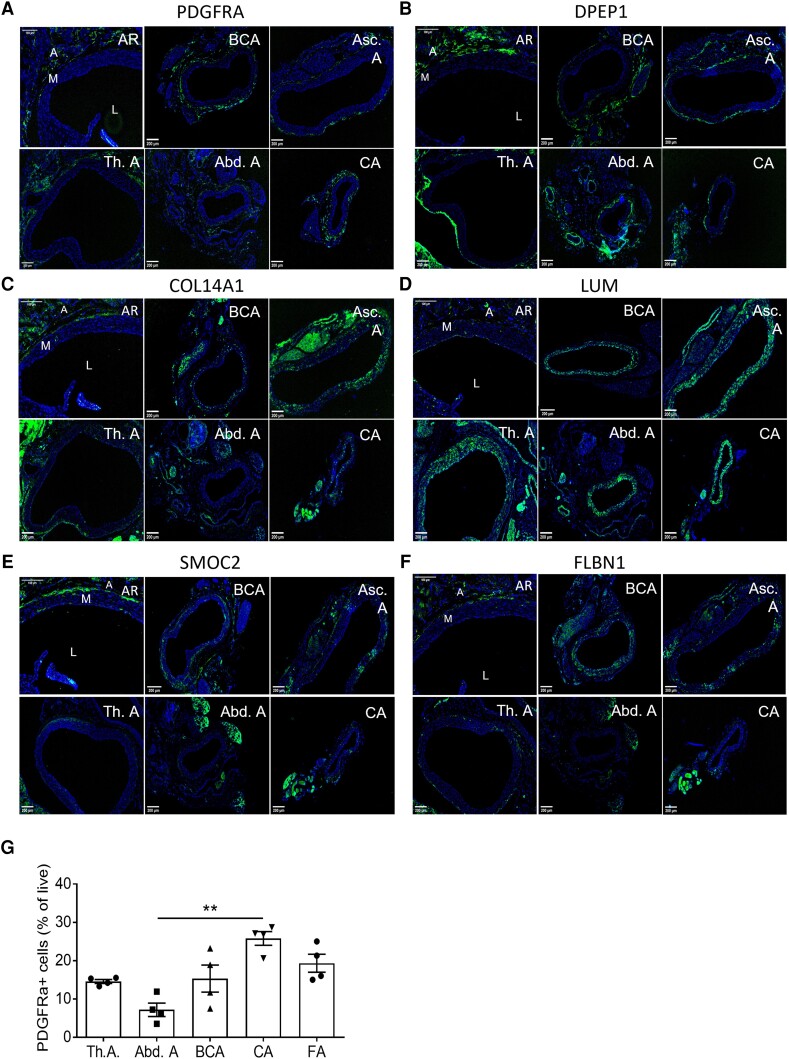
Validation of fibroblast signature across multiple vascular beds. Representative immunohistochemical staining of proposed fibroblast markers. (*A*) Platelet-derived growth factor alpha (PDGFRA, (*B*). Dipeptidase 1 (DPEP1), (*C*) Collagen 14 alpha 1 (COL14A1), (*D*) Lumican (LUM), (*E*) SPARC-related modular calcium binding 2 (SMOC2), and (*F*) Fibulin 1 (FBLN1), in healthy murine C57BL/6J aortic roots (AR), brachiocephalic artery (BCA), ascending aorta (Asc.A), thoracic aorta (Th.A), abdominal aorta (Abd.A), and carotid artery (CA) *n* = 10). Nuclei in blue and fibroblast makers in green. L indicates Lumen, M indicates media, and A indicates adventitia. (*G*) PDGFRA+ frequencies of live CD45-/CDH5+/TAGLN+ adventitial cells across C57BL/6J arteries [thoracic aorta (Th.A), abdominal aorta (Abd.A), brachiocephalic artery (BCA), carotid artery (CA), and femoral artery (FA)], analysed by flow cytometry (*n* = 4 pools of 5 mice each, 20 mice total). Statistical analysis was performed using the Kruskal–Wallis test, with Dunn’s *post hoc* test (*G*). Results are shown as mean ± standard error of the mean (SEM). *P < 0.05 vs. Th.A.

We next validated the fibroblast signature at the protein level using immunohistochemistry and confirmed adventitial localization in healthy mice and expression in spindle-like cells, resembling known fibroblast morphology for all markers, except CCL11. We used the following vascular beds: aortic root (AR), brachiocephalic artery (BCA), ascending aorta (Asc.A), thoracic aorta (Th.A), abdominal aorta (Abd.A) and carotid artery (CA) (*Figure 2*). PDGFRA and DPEP1 expression was specifically located in the adventitia across all arteries (*Figure 2A and B*), whereas LUM, SMOC2, COL14A1, and FBLN1 also showed expression in the media (*Figure 2C–F*). In case of the latter, it is in accordance with the recent detection of LUM+ myofibroblast-like cells.^12,13,28^ Negative controls can be observed in Supplementary material online, *Figure S3A*. Importantly, flow cytometry confirmed that PDGFRA expression was largely similar across various vascular beds (*Figure 2G*). CCL11 was undetectable in aortic roots (see Supplementary material online, *Figure S3B* and *C*), concordant with gene expression analyses in heart and aorta from the Tabula Muris consortium^29^ (see Supplementary material online, *Figure S1G*).

Moreover, by making use of aorta tissue from SMC myosin heavy chain 11 (*Myh11*) reporter mice and *Pdgfra* reporter mice, we were able to show very limited overlap between *Pdgfra* and *Myh11* (see Supplementary material online, *Figure S3D* and *E*). This confirmed the highly selective nature of *Pdgfra*, prompting its use in further studies to delineate fibroblast distribution across arteries and heterogeneity.

### 3.2 Trajectory inference analysis predicts the cellular dynamics of fibroblasts in healthy murine adventitia

**Figure 3:**
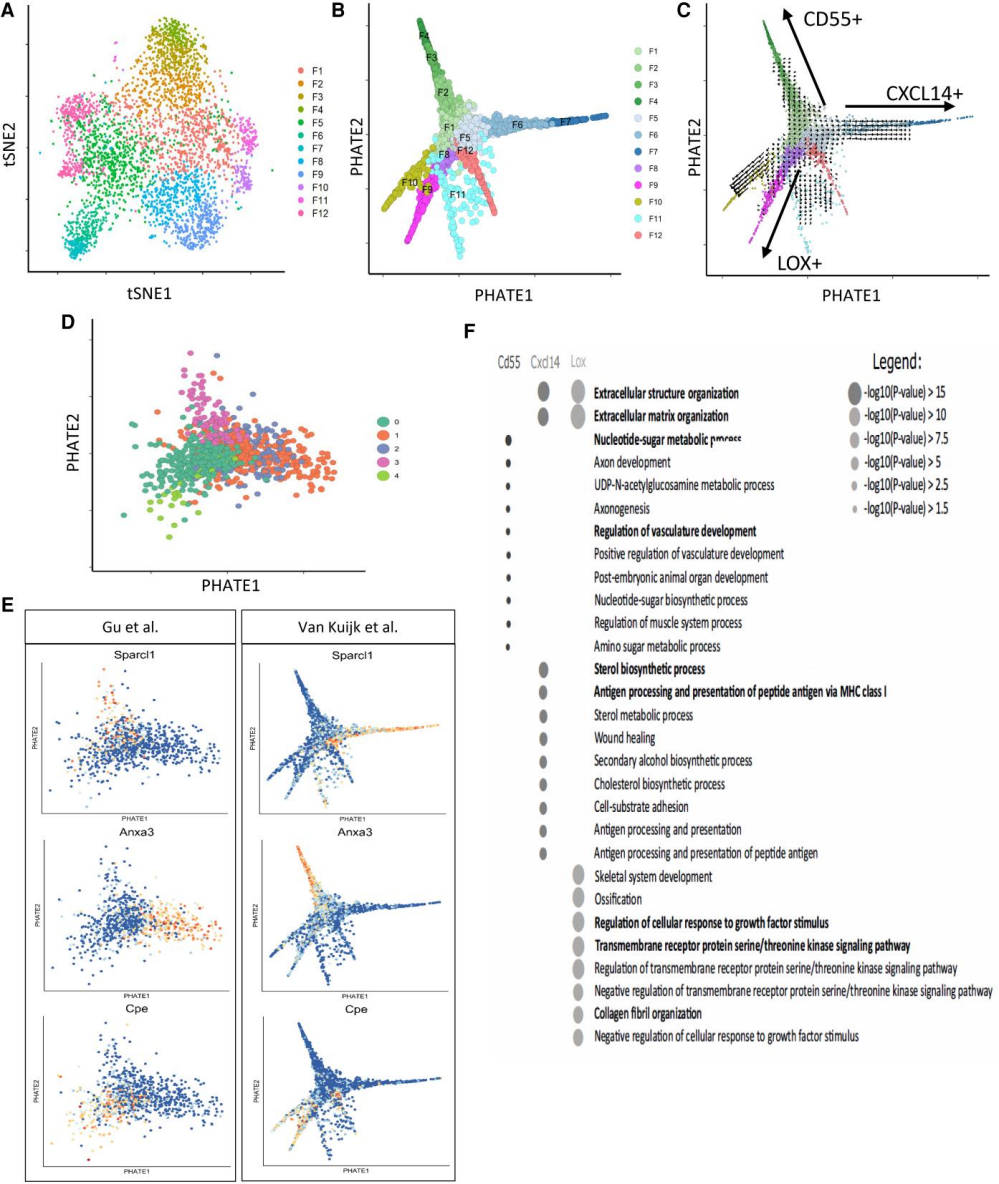
Trajectory analysis shows distinct phenotypes of fibroblasts in healthy murine adventitia. (*A*) tSNE plot of fibroblasts originating from *Figure 1D*. (*B*). PHATE pseudotime trajectory analysis of fibroblasts from *Figure 1D* depicting 12 clusters differentiating along several trajectory paths. (*C*) RNA velocity analysis on PHATE data from *Figure 3B*, arrows are indicating directionality. (*D*) Data were validated by PHATE analysis on an independent dataset from Gu *et al*.^18^ (840 cells from healthy murine adventitia) showing three trajectories. (*E*) Feature plots show the expression of three differentially expressed genes in trajectories from Gu dataset on Gu PHATE map, and their expression in three trajectories of the PHATE map of our dataset (van Kuijk). (*F*) Dot plot of the GO terms from the most differentiated clusters (F4, F7, F9) representing Trajectories 1–3, respectively, with the most relevant GO terms in bold.

The scRNA-seq analysis not only supported the existence of two distinct cell types, but also suggested heterogeneity within the fibroblast population in a healthy, basal state (*Figure 3A*). To characterize the cellular dynamics underlying fibroblast heterogeneity, we applied the Potential of Heat-diffusion for Affinity-based Trajectory Embedding (PHATE) dimensionality reduction analysis to the dataset to predict the differentiation state. PHATE reduction is developed for optimal preservation of patterns in data structure such as continual progressions, branches, and clusters, arising due to underlying biological processes, like differentiation.^23^ PHATE previously uncovered trajectories that were undiscoverable by other methods.^23^ Subsequent clustering and visualization of data revealed multiple trajectories suggestive of a continuous distinct fibroblast subtype present in the arterial wall (*Figure 3B*). Expression of stem cell marker *Sca-1/Ly6a*^30^ in most (96.5%) fibroblasts, as shown in *Figure 1F*, supports the cellular differentiation potential of these cells. Interestingly, one of the three trajectories showed higher *Sca-1/Ly6a* expression throughout the whole trajectory (see Supplementary material online, *Figure S4A*), whereas endpoint clusters of the other two trajectories did not. PHATE analysis did not predict any *Sca-1* expressing fibroblasts to be differentiated into SMCs of the healthy murine adventitia (see Supplementary material online, *Figure S4B*). To exclude that these trajectories were a result of differences in proliferation, protein synthesis, or an artefact related to cell damage, the expression of proliferation markers, and ribosomal and mitochondrial genes, respectively, were investigated. Near absent expression of proliferation markers *Mki67, Cdk1, Cdk2*, and *Cenpf*, and uniformly low expression of mitochondrial and ribosomal reads among all clusters was shown (see Supplementary material online, *Figure S4C–F*).

We next mapped RNA velocities^24^ onto the PHATE visualization. RNA velocity is estimated based on the proportions of spliced vs. unspliced transcripts, allowing for prediction of future cell transcriptional state. In agreement with PHATE analysis, vectors pointing outwards toward branch extremities suggested the differentiation direction of three main trajectories (*Figure 3C*). Application of Monocle, a third trajectory inference tool,^25^ further supported the presence of identified trajectories (see Supplementary material online, *Figure S4G*). The inference of the trajectory analysis was that all three trajectories originated from one or more clusters in the centre (F1, F5, or F8), hence the possibility of a precursor population was further investigated. Gene signatures for each of these centre clusters were constructed (see Supplementary material online, *Table S4*) and the resulting signature scores were presented in violin plots to suggest the origin of the three trajectories (see Supplementary material online, *Figure S5*). This analysis implied that the differential expression of the F1 signature in clusters F2, F3, and F4 supported F1 as the origin of this trajectory (Trajectory 1). The F1 origin of F10 and F11 is likely, but the differential expression of the F1 signature was less clear. Similarly, signature analysis suggested F5 as the likely origin of the F6–F7 (Trajectory 2) and F12 trajectories. F8 was inferred to be the likely origin of Trajectory 3 given the observed enrichment of its signature in F9.

Furthermore, the observed pattern was not a dataset-specific phenomenon, as PHATE analysis of 840 ‘non-immune’ adventitial cells in the dataset by Gu *et al.*^18^ also revealed three comparable differentiation trajectories (*Figure 3D*), supporting the results of our trajectory analysis. Expression of DEGs from the PHATE trajectories originating from the Gu dataset was also confined to three individual trajectories in our own PHATE analysis data (*Figure 3E*) demonstrating the reproducibility of our findings.

The DEGs in our dataset were further analysed to investigate possible biological traits associated with the observed trajectories. GO term analysis of DEGs identified in the distal, most differentiated clusters (i.e. F4, F7, F9) of the three trajectories revealed differential annotation of GO terms, and thus potentially different functions (*Figure 3F*). Trajectories 2 and 3 demonstrated the expression of genes involved in extracellular matrix production. Trajectory 1 showed enrichment for terms involved in vasculature development and nucleotide sugar metabolism, Trajectory 2 for cholesterol metabolism and antigen presentation, and Trajectory 3 for response and signalling upon growth factors and collagen fibril organization. Together, the analysis that supports the continuity of phenotype is apparent in adventitial fibroblasts, where most differentiated clusters have differential functional annotations.

### 3.3 Fibroblast clusters validated in healthy murine vasculature

**Figure 4:**
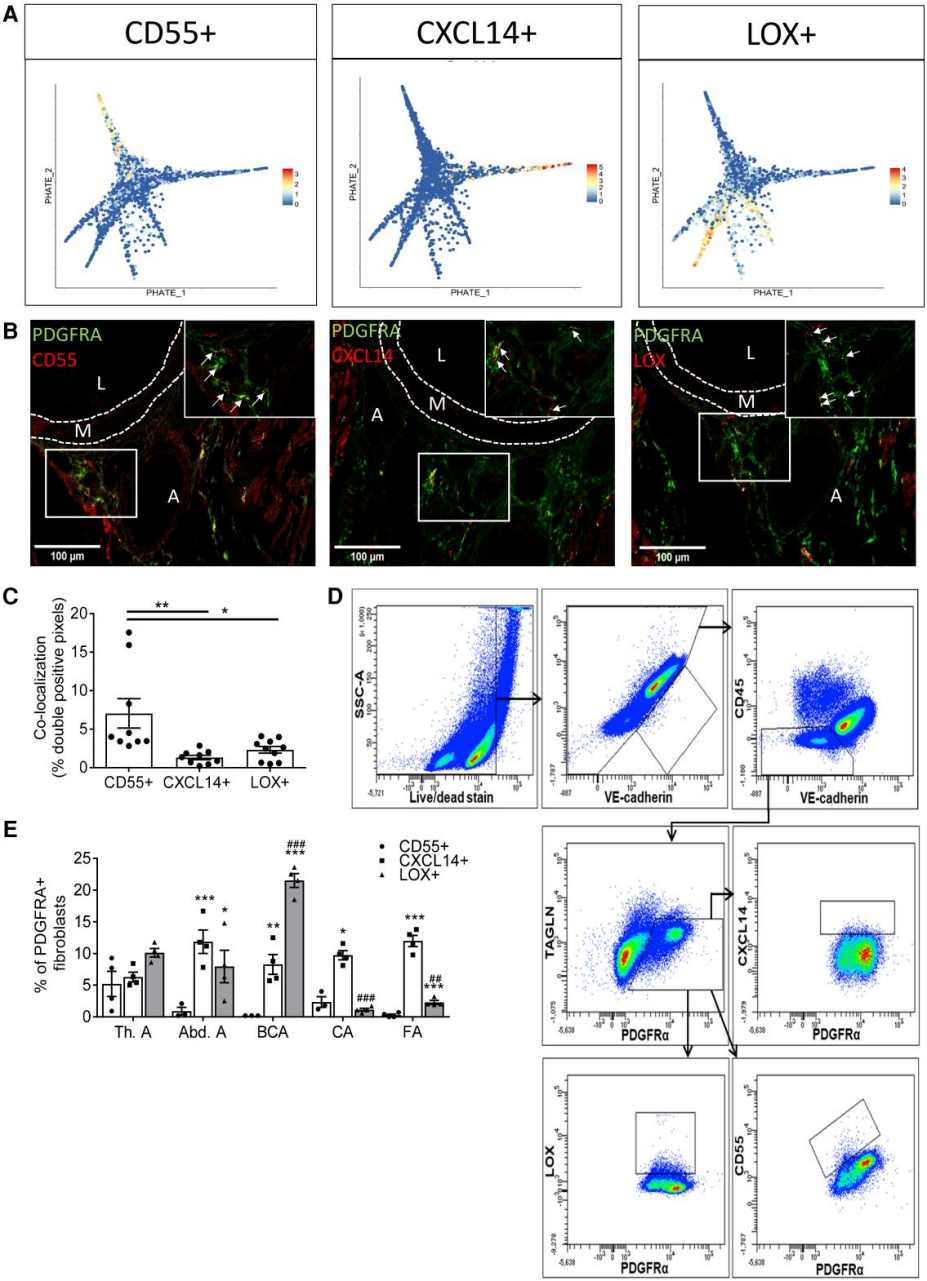
Fibroblast clusters representing three trajectories can be identified on transcriptional and protein levels in healthy murine adventitia. (*A*) Projection of cluster markers representing the three trajectories *Cd55*, *Cxcl14,* and *Lox* on PHATE plot from *Figure 3A*. (*B*) Immunohistochemical staining of CD55, CXCL14, and LOX in aortic roots of healthy C57Bl/6J mice (*n* = 10). Pan-fibroblast marker PDGFRA in green and fibroblast trajectory markers in red. Yellow areas indicate double-positive cells for PDGFRA and cluster marker (marked with arrows in 63 × magnification). L indicates Lumen, M indicates media, A indicates adventitia. (*C*) Quantification of double-positive cells for each cluster in aortic roots of *Figure 4B*. (*D*) Flow cytometry gating strategy of each fibroblast cluster. (*E*) Fibroblast clusters in the adventitia of thoracic aorta (Th.A), abdominal aorta (Ab.A), brachiocephalic artery (BCA), carotid artery (CA), and femoral artery (FA) assessed by flow cytometry in 4 pools of 5 mice, 20 mice in total. Statistical analyses were performed using one-way analysis of variance (ANOVA) with the Bonferroni *post hoc* test (*C*) or two-way ANOVA with the Tukey *post hoc* test (*E*). All results show mean ± SEM. *P < 0.05, **P < 0.01, or ***P < 0.001 vs. *CD55*+ fibroblasts in same artery; ^#^*P* < 0.05 or ^###^*P* < 0.001 vs. same cluster in Th.A.

Genes selectively marking the most differentiated cluster of each fibroblast trajectory were identified for validation at the protein level, i.e. F4, F7, and F9 for Trajectory 1 through 3, respectively (see Supplementary material online, *Figure S6*). Candidates were selected based on reported expression in fibroblasts, cellular function related to the trajectory GO terms, gene function shown in animal studies, GWAS to be related to known fibroblast functions, and/or processes involved in vascular disease, availability of antibodies for immunohistochemistry and flow cytometry, and/or preferential membrane expression. As an indicator of the most differentiated cluster in Trajectory 1, complement decay-accelerating factor (*Cd55*) (*Figure 4A*) is involved in complement activation and a whole-body KO mouse presented with a protective phenotype against atherosclerosis.^31,32^ The marker representing Trajectory 2, chemokine ligand 14 (*Cxcl14*) (*Figure 4A*), is involved in immune regulation and immune cell migration.^33^ Lastly, the marker representing Trajectory 3, *Lox*, is involved in the crosslinking and stabilization of extracellular matrix^34^ (*Figure 4A*). All three markers (CD55, CXCL14, and LOX) located to the adventitia in healthy murine aortic roots, brachiocephalic arteries, carotid arteries, and abdominal aorta, and co-localized with fibroblast marker PDGFRA (*Figure 4B and C*, Supplementary material online, *Figure S7A* and *B*). Flow cytometric analysis showed adventitial protein expression of all three markers in fibroblasts in a variety of vascular beds isolated from healthy C57BL/6J mice (*Figure 4D and E*). Important to note is that the observed percentages of each end-stage cluster in the thoracic aorta are similar to cluster percentages obtained from our scRNA-seq data (see Supplementary material online, *Table S5*). CD55+ and CXCL14+ fibroblasts are similarly present between arteries, whereas the frequency of LOX+ fibroblasts varies. All clusters show different distributions within the same artery. These data validate the location, PDGFRA co-localization, frequency, and protein expression of key markers for clusters representing each trajectory using two independent techniques.

### 3.4 CV risk factors differentially regulate fibroblast clusters

**Figure 5:**
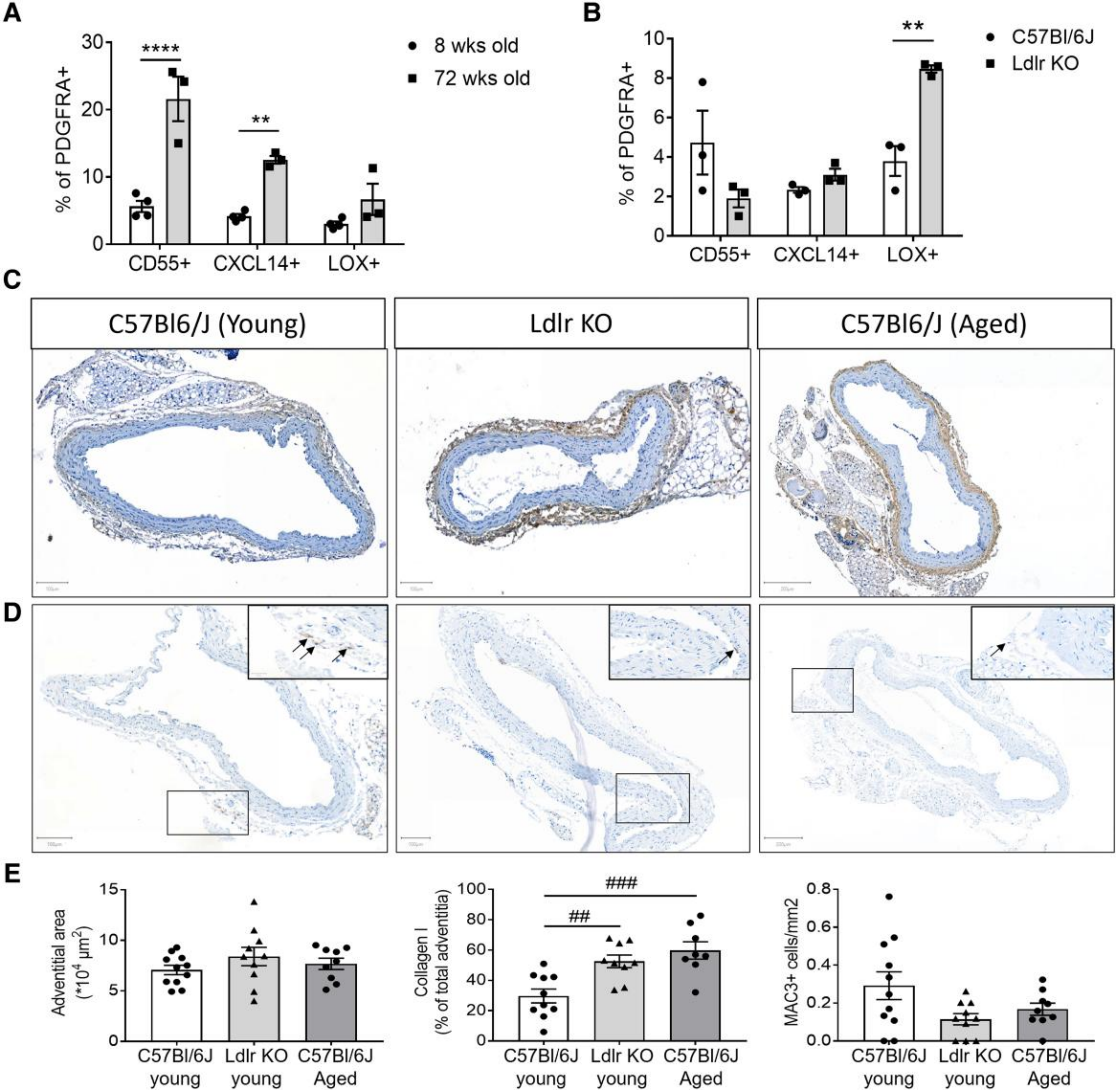
Fibroblast clusters representing three trajectories are differentially regulated upon CV risk factors. (*A*) Flow cytometry analysis of fibroblast clusters representing three trajectories in thoracic aorta adventitia from young or aged C57BL/6J mice, 12 or 72 weeks old, respectively [*n* = 4 pools of young mice, 9 mice per pool (36 mice total) vs. *n* = 3 pools of aged mice, 4–5 mice per pool (14 mice total), respectively]. Data are depicted as mean ± SEM. (*B*) Flow cytometry analysis of fibroblast clusters representing trajectories in adventitia from *Ldlr* KO mice on chow diet vs. healthy C57BL/6J mice [*n* = 3 pools, 4 mice per pool (12 mice total) vs. *n* = 3 pools, 6 mice per pool (18 mice total), respectively]. Data are depicted as mean ± SEM. (*C*) Representative images of Collagen type I, (*D*) MAC3 immunohistochemical staining, and (*E*) quantification of adventitial area, collagen type I, and MAC3+ cells in the adventitia of young, *Ldlr* KO, and aged mice (11, 10, and 9 mice per group, respectively). Positive cells or areas are observed in brown and nuclei in blue. Statistical analyses were performed using two-way ANOVA (*A* and *B*) or one-way ANOVA (*E*), with the Bonferroni *post hoc* test. All results show mean ± SEM. **P < 0.0032, ****P < 0.0001, ^##^*P* < 0.0060, ^###^*P* < 0.0006.

We next queried if the inferred trajectories would be involved in CVD and/or regulated by known CV risk factors. Indeed, we showed that DEGs from all three trajectories were significantly enriched in genes with a single nucleotide polymorphism related to CAD (see Supplementary material online, *Table S6*). Interestingly, mainly DEGs in CXCL14+ trajectory showed a highly significant enrichment and the involved DEGs could be linked to the GO terms of this trajectory, e.g. lipid metabolism and inflammation.^3,35^ Thus, we studied if changes in the environment, such as in CVD, differentially affected the most differentiated fibroblast clusters in each trajectory. The CV risk factors, ageing and mild dyslipidaemia, initiate early vascular changes and predispose to atherosclerosis, the main cause of CVD.^36^ To assess the response to these early vascular changes, we used flow cytometry to dissect changes in CD55+, CXCL14+, and LOX+ fibroblasts between young and aged mice, and between normolipidaemic wild-type mice and *Ldlr* KO mice on a chow diet to induce mild hypercholesterolaemia. Interestingly, fibroblast clusters were differentially altered upon ageing and lipidaemia. Ageing preferentially increased CD55+ PDGFRA+ and CXCL14+ PDGFRA+ cell fractions, whereas mild dyslipidaemia in *Ldlr* KO mice only increased the LOX+ PDGFRA+ cell fraction, representing the fibrosis-associated trajectory (*Figure 5A and B*, Supplementary material online, *Table S7*), suggesting the context-dependent importance of the inferred trajectories in the progression of the disease.

To interrogate whether these changes have functional relevance, we analysed adventitial area, collagen, and inflammatory cell accumulation. LOX is mainly involved in crosslinking immature collagen,^37^ and analysis of both mature collagen type I presence and Sirius Red analysis revealed an increase in mature collagen in adventitia from *Ldlr* KO mice (*Figure 5C and E*, and Supplementary material online, *Figure S8A*, respectively). Notably, the arteries in *Ldlr* KO or aged mice on chow did not show changes in the adventitial area, or the major vascular cell populations (*Figure 5C–E*, Supplementary material online, *Table S8*), or any sign of atherosclerotic plaque development compared with C57Bl/6J, as expected in only mild hypercholesterolaemia and ageing (*Figure 5C and D*). Immune cell infiltration did not associate with CD55+ or CXCL14+ fibroblasts in ageing. Yet, CXCL14+ fibroblasts, also predicted to act in matrix metabolism, emerged simultaneously as adventitial collagen accumulation in ageing. Hence, the functional changes coinciding with an increase of LOX+ or CXCL14+ fibroblasts precede overt inflammatory vascular disease.

### 3.5 Atherosclerosis relevance of murine fibroblast clusters and trajectories

**Figure 6:**
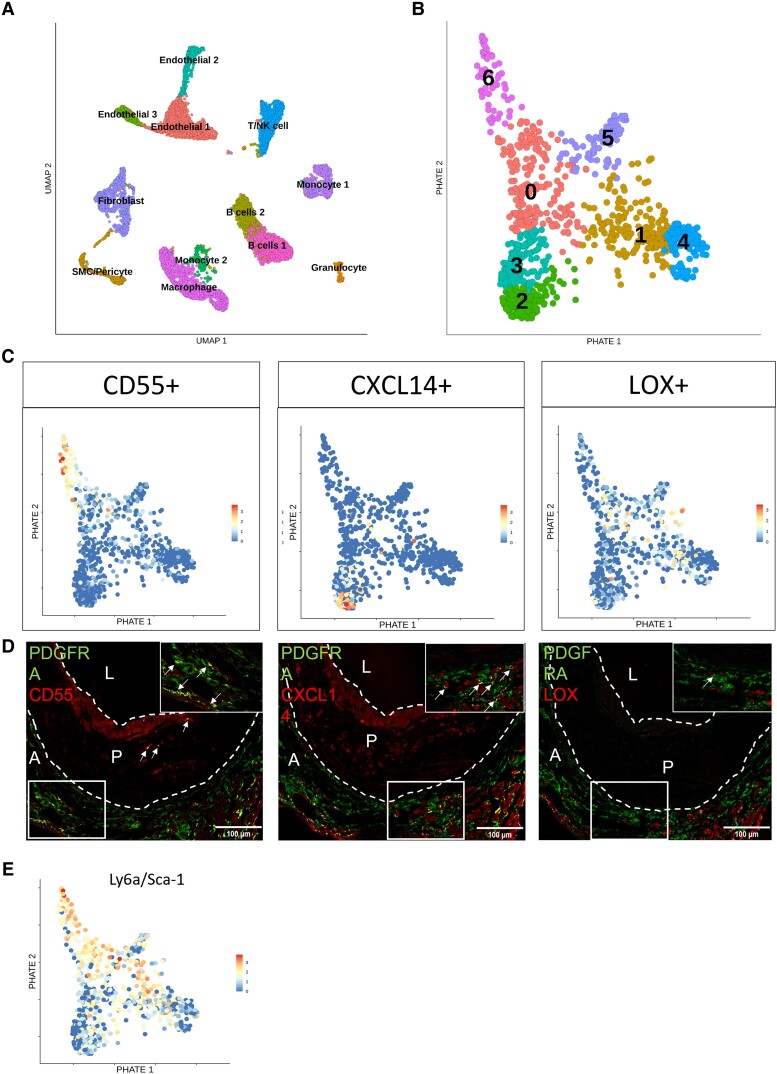
Fibroblast cluster markers representing three trajectories are still observed in atherosclerosis, while LOX+ fibroblasts reduced in presence. (*A*) Unsupervised clustering of single-cell sequencing data from *Ldlr* KO mice on chow or 16 weeks of HCD. Results are visualized by Uniform Manifold Approximation and Projection (UMAP), colours represent individual clusters. (*B***)** PHATE visualization of fibroblasts originating from the dataset in *Figure 6A*, colours represent individual clusters. (*C*) Cluster markers projected on fibroblast PHATE plot of *Figure 6B*, representing Trajectory 1 using *Cd55*, Trajectory 2 using *Cxcl14*, and Trajectory 3 using *Lox*. (*D*) Protein expression of each cluster marker visualized by immunohistochemistry in aortic roots from *Ldlr* KO mice after 16 weeks HCD. Pan-fibroblast markers in green and fibroblast cluster markers in red. Yellow areas indicate double-positive cells for pan fibroblast and cluster marker (marked with arrows). L indicates Lumen, P indicates plaque, A indicates adventitia. (*E*) *Sca-1/Ly6a* mRNA expression visualized on PHATE map, originating from *Figure 6B*, depicting fibroblast clusters.

The differential regulation by early vascular changes prompted us to study the response of adventitial fibroblast clusters to atherosclerosis using scRNA-seq transcriptomics of the adventitia in mild and severe hypercholesterolaemic *Ldlr* KO mice. In chow-fed mice, 4800 adventitial cells passed quality control and in HCD-fed mice, almost 8000 adventitial cells passed the quality control (see Supplementary material online, *Tables S1* and *S2*). All expected major cell types in adventitia were identified, with sub-clustering of the identified fibroblast population revealing seven distinct clusters (*Figure 6A and B*, Supplementary material online, *Figure S8B* and *C*). Of note, fibroblast Ly6a/Sca-1 expression was lower in disease, in line with variation in other datasets (see Supplementary material online, *Table S9*). PHATE reduction analysis confirmed the presence of trajectories equivalent to the original three trajectories in the healthy adventitia (*Figure 6C*). Expression patterns of *Cd55* and *Cxcl14* each remained confined to a single fibroblast trajectory (*Figure 6C*). This was to a lesser extent visible for *Lox*. *Lox* was less confined to one trajectory, although still mutually exclusive from cells expressing *Cd55*+ *Cxcl14*+. In line with mRNA expression patterns, protein expression of markers for all three trajectories was visualized in PDGFRA fibroblasts of the adventitia underlying advanced murine plaques (*Figure 6D*). LOX+ fibroblasts were the least prominent at the protein level in this disease condition. These data imply a role for LOX+ fibroblasts in the very early stages of atherogenesis, rather than advanced atherosclerosis.

Interestingly, only CD55+ fibroblasts were observed in the atherosclerotic plaque, indicated by the white arrows, in addition to the adventitial layer (*Figure 6D*). Intriguingly, this trajectory (Cluster 0 and 6) also highly expressed stem cell marker *Sca-1/Ly6a* (*Figure 6E*) and may represent the most plastic, progenitor-like trajectory. This is in line with our healthy scRNA-seq dataset, where the equivalent trajectory highly expressed *Sca-1/Ly6a*. Other groups have already shown that SCA-1 positive cells have the capacity to contribute to neointima formation upon vascular injury,^38,39^ yet it remains to be defined if these cells were of fibroblast, MC, or other origin. Our data shed new light on the possible role of specific fibroblast trajectories therein.

### 3.6 Fibroblast clusters are present in atherosclerotic human vasculature

**Figure 7:**
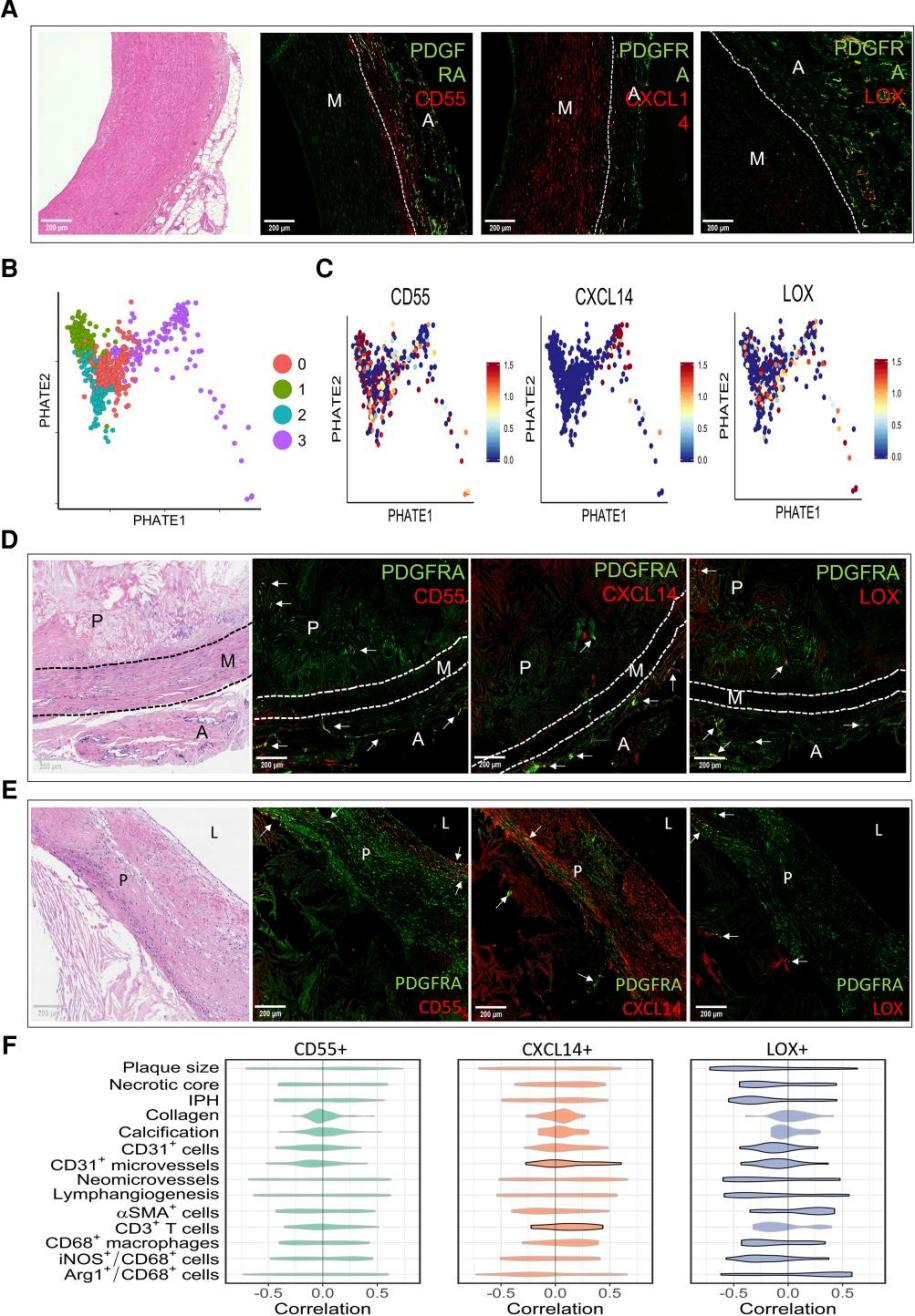
Fibroblast trajectories correlate differentially to human atherosclerotic plaque phenotype. (*A*) Immunohistochemical staining of CD55+ fibroblasts, CXCL14+ fibroblasts, and LOX+ fibroblasts representing Trajectories 1–3, respectively, in human IT specimens collected through autopsy, accompanied with corresponding H&E, pan-fibroblast marker in green, and fibroblast trajectory markers in red. Yellow areas indicate double-positive cells for pan fibroblast and cluster marker. M indicates media and A indicates adventitia. (*B*) PHATE analysis of fibroblasts in scRNA-seq dataset by Li *et al*.^19^ showing four clusters. (*C*) Fibroblast cluster markers representing the trajectories from mouse scRNA-seq data extrapolated to feature plots of human control data. Immunohistochemical staining of CD55+ fibroblasts, CXCL14+ fibroblasts, and LOX+ fibroblasts representing Trajectories 1–3, respectively, in advanced human atherosclerotic plaques, showing the adventitial side (*D*) and the luminal side (*E*), accompanied by the corresponding H&E. Pan-fibroblast marker in green and fibroblast trajectory markers in red. Yellow areas indicate double-positive cells for pan fibroblast and cluster marker. M indicates media, P indicates plaque, A indicates adventitia. (*F*) Violin plots depicting correlations of all genes differentially expressed by each fibroblast trajectory with plaque traits in 43 human carotid plaque segments. Significant violin plots (*P* < 0.05) were denoted with a black border. Significance was assessed by positive and negative correlations and the unbalance thereof, which was defined as the sum of positive correlations minus the sum of absolute values of negative correlations. Furthermore, correlation skewness was compared between trajectory genes and a random gene set containing a similar number of genes. The permutation test was performed 100 000 times and the *P*-value is the frequency of the random gene sets that have higher correlation skewness than the trajectory gene set.

To address the relevance of our murine fibroblast trajectories in human vasculature, we used specimens from carotid anastomosis during aortic bypass surgeries and carotid artery specimens acquired from the opposite side of the culprit plaques during carotid endarterectomy. Both specimens have the advantage that the adventitia is still attached to the vessel wall, allowing investigation of the trajectories in very early stage atherosclerotic human adventitia. Healthy specimens are almost impossible to retrieve in the western population, as even asymptomatic patients present with the earliest signs of intimal thickening (IT).^40^ This precludes the use of completely healthy arteries, as we obtained from mice. Nevertheless, all cluster markers representing the three trajectories could be observed in the adventitia of both surgical specimens (*Figure 7A*, Supplementary material online, *Figure S9A*), ensuring the biological relevance of our identified clusters in human vasculature. In addition, in IT specimens obtained through autopsy from patients without CV symptoms, clusters could also be observed in the adventitia (see Supplementary material online, *Figure S9B*). Moreover, spatial location might be of importance for function. In human IT sections, CD55+ fibroblasts were often observed on the border of the adventitia and media, whereas CXCL14+ and LOX+ trajectories were more observed surrounding the blood vessels in the adventitia.

To further confirm the presence of trajectories in human vasculature with early signs of disease, we obtained aorta scRNA-seq data from elderly individuals (median age 62) including all arterial wall layers.^19^ As these subjects presented with a history of smoking (*n* = 2), diabetes mellitus (*n* = 1), or hypertension (*n* = 1), aortae morphology is expected to show early signs of disease. After the selection of the fibroblasts in the dataset, we performed PHATE analysis to assess the presence of trajectories. Also, in human aorta with early atherosclerosis, trajectories could be observed that were transcriptionally divergent, although to a lesser extent than in young, healthy mouse adventitia (*Figure 7B*). Our murine cluster markers were expressed in human aorta fibroblasts, whereas only *CXCL14* was strictly confined to one human trajectory (*Figure 7C*). As this is a simplified view based on one marker gene, we tested if the compete gene set differentially expressed by each murine trajectory was significantly enriched in human fibroblasts. Important to note is that genes of the murine trajectories were indeed significantly enriched in the human fibroblasts (see Supplementary material online, *Table S10*). Together these data support the human relevance of the observed fibroblast heterogeneity in mice.

We additionally confirm the presence of the fibroblast clusters in advanced human atherosclerotic plaques of symptomatic patients undergoing carotid endarterectomy. Protein expression of each cluster marker was confirmed in adventitial PDGFRA+ fibroblasts, but also in the advanced plaque itself (*Figure 7D and E*) both on the adventitial and the luminal side. Additionally, we correlated differentially expressed genes by murine *CD55*+, *CXCL14*+, and *LOX*+ fibroblasts (46, 32, and 23 genes, respectively) to human plaque traits.^21^ The traits were quantified in histology sections adjacent to the segment used for transcriptomics. The distribution of the individual correlations for all genes in a particular fibroblast cluster is shown in *Figure 7F*. Mostly genes of LOX+ fibroblasts were shown to negatively correlate with detrimental plaque traits, such as plaque size, necrotic core, and inflammatory macrophages (*Figure 7F*). These data suggest differential regulation and/or functions of fibroblast clusters representing the trajectories in human atherosclerosis, as we observed in mice.

## 4. Discussion

In this study, we identified arterial fibroblast cell-type markers *Pdgfra* and *Dpep1* as the most robust and unveiled pseudotime trajectories of CD55+, CXCL14+, and LOX+ fibroblasts on RNA and protein level across five independent RNA datasets and using histology of five different murine and human arteries. We provide biological implications of these fibroblast clusters in disease in mice and humans: (i) CV risk factors and concomitant environmental triggers drive differential cluster distribution and associate with adventitial fibrosis; (ii) ageing regulated adventitial CD55+ and CXCl14+ fibroblast expansion and collagen accumulation; (iii) mild hypercholesterolaemia stimulated LOX+ fibroblast expansion and adventitial fibrosis preceding atherosclerosis; (iv) fibroblast trajectories are present in human adventitia and plaques of symptomatic patients, (v) fibroblast trajectory genes differentially associated with human plaque traits and were enriched in GWAS genes, suggesting functional implication in human disease development. Together, these findings demonstrate a functional role for adventitial fibroblast trajectories, which could be of interest in disease progression and thus targeted treatments.

The identified arterial fibroblast cell-type signature is of importance to the field to accurately distinguish arterial fibroblasts from other vascular cells, as the expression of traditional fibroblast markers (e.g. COL1A1/2, VIMENTIN, CD90, S100-A4, FAP, and DCN) is generally not restricted to fibroblasts as shown here and by others.^11^ Despite extensive *in silico* validation in three other single-cell transcriptomics datasets in healthy vasculature, protein validation only supported adventitial specificity of PDGFRA and DPEP1 across vascular beds, whereas LUM, COL14A1, SMOC2, and FBLN1 were additionally expressed in the media. Presumably, markers are shared with medial SMCs, in line with the recent identification of LUM as the marker for dedifferentiated SMCs in disease.^13^ This is important information, as LUM has been coined as a fibroblast marker in several single-cell studies with mouse, primate, and human arteries, yet without proper validation.^17,41,42^ Alternatively, differences in embryonic origin between arteries could explain medial expression, in line with different embryonic origins of SMC.^43^ The embryonic origin of adventitial fibroblasts in most arteries is not fully clear but is important to understand homoeostasis and response to injury. Previous work showed that the neural crest was the origin of coronary artery adventitia,^43^ yet others excluded this origin in ascending aorta and support second heart field.^44^ Instead, dedifferentiation of medial SCA-1/LY6A+ SMCs was shown,^27^ which offers a third explanation of ambiguity of our fibroblast signature. Transdifferentiation between SMCs and fibroblasts in atherosclerosis is seemingly bi-directional.^13,45^ Our data indeed suggest variation in embryonic origin and/or transdifferentiation across arteries. Whether this also explains variation in trajectory dominance across arteries remains to be resolved using dual lineage reporter mice. Overall, the adventitial-specific location of PDGFRA and DPEP1 across arteries and absent medial co-localization of PDGFRA and SMC marker MYH11 in lineage reporter mice support the specificity of this marker for arterial fibroblasts across healthy arteries, recommending this marker for future studies. A *Pdgfra* lineage tracing mouse would give insight into the location and distribution of fibroblasts healthy but also diseased adventitia. In atherosclerosis, this would also reveal the fate of adventitial fibroblasts, which is of interest considering evidence of endothelial or SMC origin of fibroblast-like cells in plaques.^13,14^ These studies are, however, beyond the scope of the current study.

The importance of adventitial cells in vascular pathology has been studied over the years, specifically focusing on the Ly6aSca-1+ progenitor population as a whole,^6,45^ as recently reviewed by Jolly *et al*.^1^ This population includes both mesenchymal and immune progenitors as shown by targeted phenotyping, and by our own data. Using our unbiased approach to phenotype adventitial mesenchymal cells, we show that the *Pdgfra/Dpep1* fibroblast population includes *Ly6a/Sca-1*+ cells, but also *Ly6a/Sca-1* low or negative cells. Moreover, *Ly6a/Sca-1*+ fibroblast decreases in the presence of atherosclerosis, which might be a result of differentiation upon disease induction. On the other hand, we show that adventitial LY6A/SCA-1+ cells include more than fibroblasts alone. Hence, *Ly6a/Sca-1*+ cells do not fully recapitulate PDGFRA+ cells, a concept which is important for the interpretation of results. The CD55+ trajectory cells express a high level and frequency of *Ly6a/Sca*-1 and its function may thus most closely resemble published reports on adventitial *Ly6a/Sca-1*+ progenitor cells.

Biological implications of CD55+, CXCL14+, and LOX+ fibroblasts may be gained from their differential association and response to experimentally changed CV risk factors, i.e. age and serum lipids, and enrichment of genes with a GWAS to CAD. CD55+ fibroblasts were linked to vascular development and were increased upon ageing. In endometrioid tumour, CD55 was found to be essential in self-renewal,^46^ which would be in line with our findings of coinciding expression of Sca-1 and CD55+ trajectory. Increasing the presence of the CD55+ trajectory might induce rejuvenation through increased plasticity and potential to adapt to pathogenesis. In addition, CD55 has a role in complement regulation, and its stimulation may trigger detrimental vascular inflammation. This is in line with observation in atherosclerosis, where whole-body CD55 deficiency was shown to be atheroprotective in ApoE KO mice.^31^ As CD55 is one gene of 46, skewing the entire trajectory would probably not reflect the effect of the single CD55 knock-out. CXCL14+ trajectory also expanded upon vascular ageing. GO terms of the CXCL14+ trajectory included extracellular matrix organization and antigen presentation, among others. In vascular ageing, we only observed an association of this trajectory with fibrosis, likely owing to the four collagen genes in this trajectory (*Col4a1, 5a3, 6a3, 15a1*). This is in line with a positive effect on fibrotic gene expression and proliferation of fibroblasts,^47^ and the absent effect of *Cxcl14* KO on immune cell recruitment in homoeostasis.^48^ However, upon a stronger pro-inflammatory milieu, like in overt atherosclerosis, this aspect of CXCL14 function may be important. Indeed, this trajectory was also detected in advanced plaques by histology and single-cell sequencing. In line, *Cxcl14* expression was enhanced in mouse primary macrophages by oxidized LDL, and peptide immune therapy diminished serum CXCL14 levels and murine atherosclerosis.^49^ Although attributed to macrophages so far, conditional deletion of *Cxcl14* using existing *Pdgfra-* or future *Dpep1*-Cre models may unveil the effect of CXCL14+ fibroblasts in atherogenesis.

While CD55+ and CXCL14+ fibroblasts expanded upon vascular ageing, the expansion of LOX+ fibroblasts was triggered only by a mild increase in serum cholesterol. The early rise of LOX+ fibroblasts coinciding with adventitial collagen deposition prior to disease development possibly implies a regenerative role for LOX+ fibroblasts to strengthen the vessel upon a lipid challenge. Higher total LOX protein abundance in plaques was associated with plaque stability, whereas, seemingly opposing, *Lox* mRNA levels predicted the risk of myocardial infarction.^50^ Although these effects of LOX have thus far been attributed to SMCs,^51^ future studies are warranted to challenge this view. Together, we foresee skewing trajectories towards more favourable subsets through conditional knock-out models, which might have great relevance for atherogenesis and vascular ageing, like the improved balance between lung myogenic and lipofibroblasts spurring lung fibrosis.^52^ Likewise, dampening pro-inflammatory fibroblasts or promoting matrix fibroblasts may be beneficial for plaque progression. An interesting addition to this is that lipid-lowering medications that are prescribed on a regular basis, e.g. statins, could already influence fibroblast abundance and matrix production.^53^ Studies investigating the beneficial lipid-lowering effect vs. the negative effect on fibroblast presence and functions are warranted.

The current study has some limitations. Current single-cell sequencing technology has limited sequencing depth and is, therefore, biased towards genes with high expression levels. Nevertheless, the resolution at the single-cell level has already provided new insights into arterial biology in health and disease, as well as corroborated existing ones.^2,18,27,54^ Enrichment of mesenchymal cells yielded sufficiently high fibroblast cell numbers to reveal transcriptional regulation of small subsets of cells, which remained obscured in two ‘atlas’ datasets with smaller fibroblast numbers.^17,18^ While their approach had the advantage to study all cells simultaneously, as well a cell–cell communication, our approach prevents analysis of cell–cell communication. Moreover, the lack of healthy, human adventitial single-cell sequencing datasets prevents direct comparison of the adventitial fibroblast transcriptome and subsets between mice and humans. Another limitation pertains to a causal implication of the observed association between the fibroblast trajectories and human plaque characteristics. Future studies with conditional depletion of trajectory genes or their master regulators in *Pdgfra+/Dpep1*+ fibroblasts would give us insight into how targeted elimination of fibroblast trajectories would impact atherogenesis.

In conclusion, PDGFRA specifically marks arterial fibroblasts across arterial beds, with CD55+, CXCL14+, and LOX+ fibroblasts showing differential association to human CVD and response to CV risk factors. Together, these new insights will aid to determine the role of fibroblasts in disease progression and future targeted treatment plans.

## Supplementary material

Supplementary material is available at *Cardiovascular Research* online.

## Authors’ contributions

J.C.S., A.H.B, K.V.K., I.R.M.: conceptualization; K.V.K., I.R.M., R.J.H.A.T., S.E.J.A., R.W.S., R.S.T. A.H.B., and J.C.S.: methodology; K.V.K., I.R.M., R.J.H.A.T., S.E.J.A., R.W.S., R.S.T., and J.C.S.: formal analysis; K.V.K., I.R.M., R.J.H.A.T., S.E.J.A., R.W.S., R.S.T., C.K., H.J., S.M., D.K., M.J.G., L.T., J.L., P.G.: investigation; R.D., P.R., Y.L., H.N., J.R.W.K., L.J.S., Y.H.S., B.M.E.M., E.A.L.B., N.C.H., and R.K.: resources; J.C.S. and K.V.K.: writing—original draft; K.V.K., I.R.M., A.H.B., J.C.S.: writing—review and editing; J.C.S.: funding acquisition; A.H.B., J.C.S.: supervision.

## Supplementary Material

cvad016_Supplementary_Data

## Data Availability

Data are deposited (GSE196395) and may be inspected on a web-based interface (Plaqview.com).^55^ Count matrices and codes are available upon reasonable request.

## Notes

Translational perspective The current study sheds light on the transcriptional heterogeneity of arterial fibroblasts and the influence of pathologic environments, such as lipid levels or ageing, on fibroblast differentiation through transcriptional trajectories. In addition, the presence of fibroblast clusters in human specimens and their enrichment of genes associated with cardiovascular disease (CVD) highlight their potential role in disease development and progression. Novel therapeutic strategies could be shifting the focus to cell-specific targeting. By influencing fibroblast differentiation, their functional properties could be altered as well, e.g. adapting pro-fibrotic potential. This could be of great importance in regulating the effect of fibroblasts on CVD.
