# Supplementary material for: Human and murine fibroblast single-cell transcriptomics reveals fibroblast clusters are differentially affected by ageing and serum cholesterol
Source: Cardiovasc Res. 2023 Jan 31;119(7):1509–23. doi: 10.1093/cvr/cvad016 (PMC10318398; doi:10.1093/cvr/cvad016)
Supplement: cvad016_Supplementary_Data [file cvad016_supplementary_data.zip › Suppl info_Adv fib_van Kuijk_20-9-22.pdf]

<u>Online data supplement to:</u>

Human and murine fibroblast single cell transcriptomics reveals reveals fibroblast clusters are differentially affected by ageing, and serum cholesterol

<u>Fibroblast clusters in health and disease</u>

van Kuijk K[1,2], McCracken IR[3], Tillie RJHA[1], Asselberghs SEJ[1,4], Kheder DA[1], Muitjens S[1], Jin H[1], Taylor RS[3], Wichers Schreur R[1], Kuppe C[2,5], Dobie R[6], Ramachandran P[6], Gijbels MJ[1,7,8], Temmerman L[1], Kirkwoord PM[6], Luyten J[1,4], Li Y[9,10], Noels H[11], Goossens P[1], Wilson-Kanamori JR[5], Schurgers LJ[1,2], Shen YH [9,10], Mees BME[1,4], Biessen EAL[1,11], Henderson NC[5,12], Kramann R[2,4], Baker AH[1,3], Sluimer JC[1,3]

<u>Table of contents:</u>

Supplemental tables S1-10 (page 2)
Supplemental figures S1-9 (page 12)
Supplemental Video 1 legend (page 21)
Supplemental methods (page 22)
Supplemental references (page 26)

Supplemental Tables

Supplemental Table S1. Cell counts after quality control as indicated in CellRanger software

Parameter	C57BL/6J (#)	*Ldlr* KO, chow (#)	*Ldlr* KO, 16 weeks HCD (#)
Estimated number of cells	5,701	4,822	7,989
Mean reads/cell	87,456	63,948	47,390
Median genes/cell	2,490	2,359	1,640
Median UMI counts/cell	7,169	6,794	3,545

Supplemental Table S2. Sequencing parameters for 10X Genomics samples

Sequencing	C57BL/6J (%)	*Ldlr* KO, chow (%)	*Ldlr* KO, 16 weeks HCD (%)
Valid barcodes	98.4	96.0	95.8
Reads mapped confidently to transcriptome	66.9	51.6	44.4
Reads mapped confidently to exonic regions	69.3	56.0	48.5
Reads mapped confidently to intronic regions	15.1	30.8	36.0
Reads mapped confidently to intergenic regions	2.8	4.3	5.0
Sequencing saturation	85.4	69.0	65.0

Supplemental Table S3. Expression of markers macrophages, endothelial cells, neurons and adipocytes in C57BL/6J scRNA-seq dataset

Markers	Cells with detected expression (%)
Cd68 (Macrophages)	0.25
Pecam1 (Endothelial cells)	6.13
Rbfox3 (Neurons)	1.45
Adipoq (Adipocytes)	0.50

Supplemental Table S4. Differentially expressed genes (DEGs) in each individual core cluster compared to the other core clusters (F1 vs. F5 vs. F8)

F1 vs. F5 & F8 signature	F5 vs. F1 & F8 signature	F8 vs. F1 & F5 signature
Pla1a	Cxcl12	Mfap4
Gm12840	Gdf10	Col8a1
Ifi205	Steap4	Cilp
Sult1e1	Nrp1	Eln
Lrrn4cl	Ccl11	Angptl1
Ifi204	Clec11a	Fxyd6
Wt1		Itgbl1
Cotl1		Aspn
Efemp1		Sfrp1
		Lox
		Tgfb3
		Cpxm2
		Pdgfrl
		Wisp2
		C1qtnf2
		Fgl2
		Avpr1a
		Pcsk5
		Pmepa1
		Fibin
		Dkk2
		Hmcn2
		Crispld2
		Cpe
		Cdkn1c

Supplemental Table S5. Fibroblast cluster proportions represented as the percentages of the total population of fibroblast.

Cluster	F1	F2	F3	F4	F5	F6	F7	F8	F9	F10	F11	F12
**Proportion (%)**	16.54	9.26	8.73	3.83	17.21	5.27	3.75	10.60	8.59	3.02	3.24	9.96

Supplemental Table S6. Enrichment of differentially expressed genes of each trajectory in GWAS for coronary artery disease (CAD) expressed as p-value.

GWAS CAD	p-value	Intersected genes
**CD55+ Trajectory (F1234)**	$3.5*10^{-2}$	IL6R
**CXCL14+ Trajectory (F567)**	$<5*10^{-6}$	LPL, LOXL1, WT1, SERPINH1, COL6A3
**LOX+ Trajectory (F89)**	$2*10^{-3}$	TMEM204, GEM, ZEB2

Supplemental Table S7. Blood cholesterol levels of young C57BL/6J mice, aged C57BL/6J mice and *Ldlr* KO mice on chow diet or high cholesterol diet for 16 weeks

Mouse model	Diet	Cholesterol levels (mmol/L)
C57BL/6J (N=36)	Chow (8 wks old)	1.30±0.55
C57BL/6J (N=14)	Chow (72 wks old)	1.28±0.62
Ldlr KO (N=11)	Chow	4.10±2.25
Ldlr KO (N=13)	16 weeks high cholesterol diet	14.23±8.33

Supplemental Table S8. Adventitial cell populations of *Ldlr* KO mice on chow diet vs. normolipidemic C57BL/6J mice, proportional to relevant populations measured by flow cytometry. Values are represented as average from 3 pools, consisting of 3-6 mice.

Mouse model	Endothelial cells (% of living)	Immune cells (% of living, VE-cadherin-)	Smooth muscle cells (% of living, VE-cadherin-,CD45-)	Fibroblasts (% of living, VE-cadherin-,CD45-)	Other (% of living)
C57BL/6J (N=36)	0.2	1.2	3.0	31.0	64.6
Ldlr KO (N=11)	0.8	0.8	3.8	26.5	68.1

Supplemental Table S9. Percentages of Ly6a/Sca-1 expressing fibroblasts in single cell sequencing datasets used in the current manuscript.

Data	Publication	% of fibroblasts expressing Ly6a/Sca-1
**Healthy C57BL/6J**	This manuscript	94.8
**Ldlr KO (Chow)**	This manuscript	92.9
**Ldlr KO (HCD)**	This manuscript	59.3
**C57BL/6J**	Gu et al. 2019 ATVB	86.0
**C57Bl/6**	Dobnikar et al. 2018 Nature Communications	43.6

Supplemental Table S10. Enrichment of murine trajectory-specific genes in human fibroblast population originating from dataset by Li et al.

Trajectory	-10log(p-value)
**CD55+ Trajectory (F1234)**	2.17
**CXCL14+ Trajectory (F567)**	15.32
**LOX+ Trajectory (F89)**	21.24

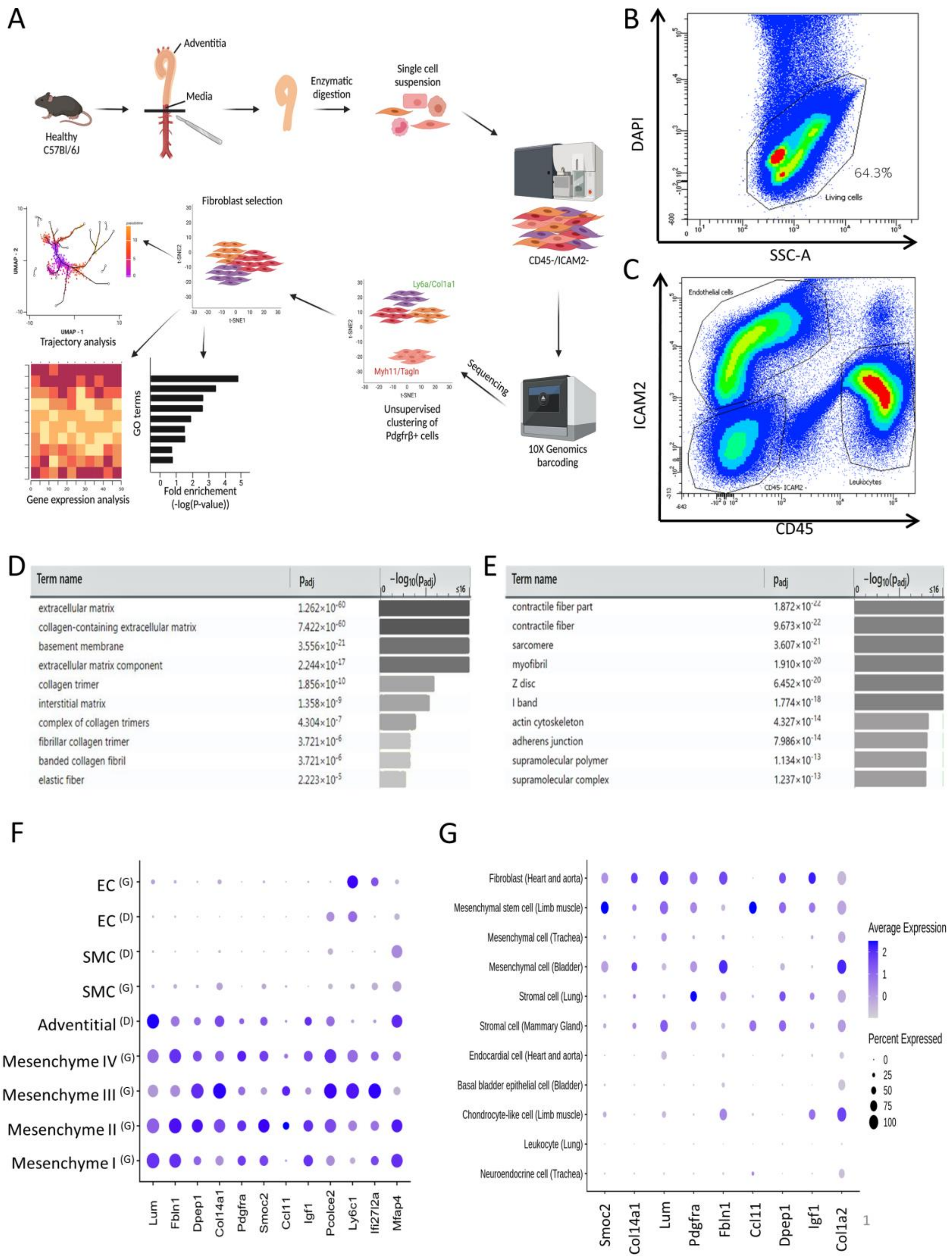

Supplemental figure S1. Fibroblast characterization using single cell sequencing. A. Diagram depicting study approach from tissue isolation from healthy C57Bl/6J mice and further processing, **B.** Selection of living DAPI- cells (64.3%) from pooled adventitial samples of 8 male mice **C.** Flow cytometry gating strategy for selection of CD45-, ICAM2- cells from DAPI- cells (9.7% of living) from figure 1B for scRNA-seq, **D.** Top ten GO-term analysis of cellular processes of fibroblasts, or **E.** MCs. **F**. Dot plot of marker specificity in healthy murine adventitia (G)[56] , and healthy media (D) [57], **G.** Marker validation in mesenchymal and fibroblasts from single cell expression data of Tabula Muris consortium [58]. Annotation of cell types in **F** and **G** is according to the original paper.

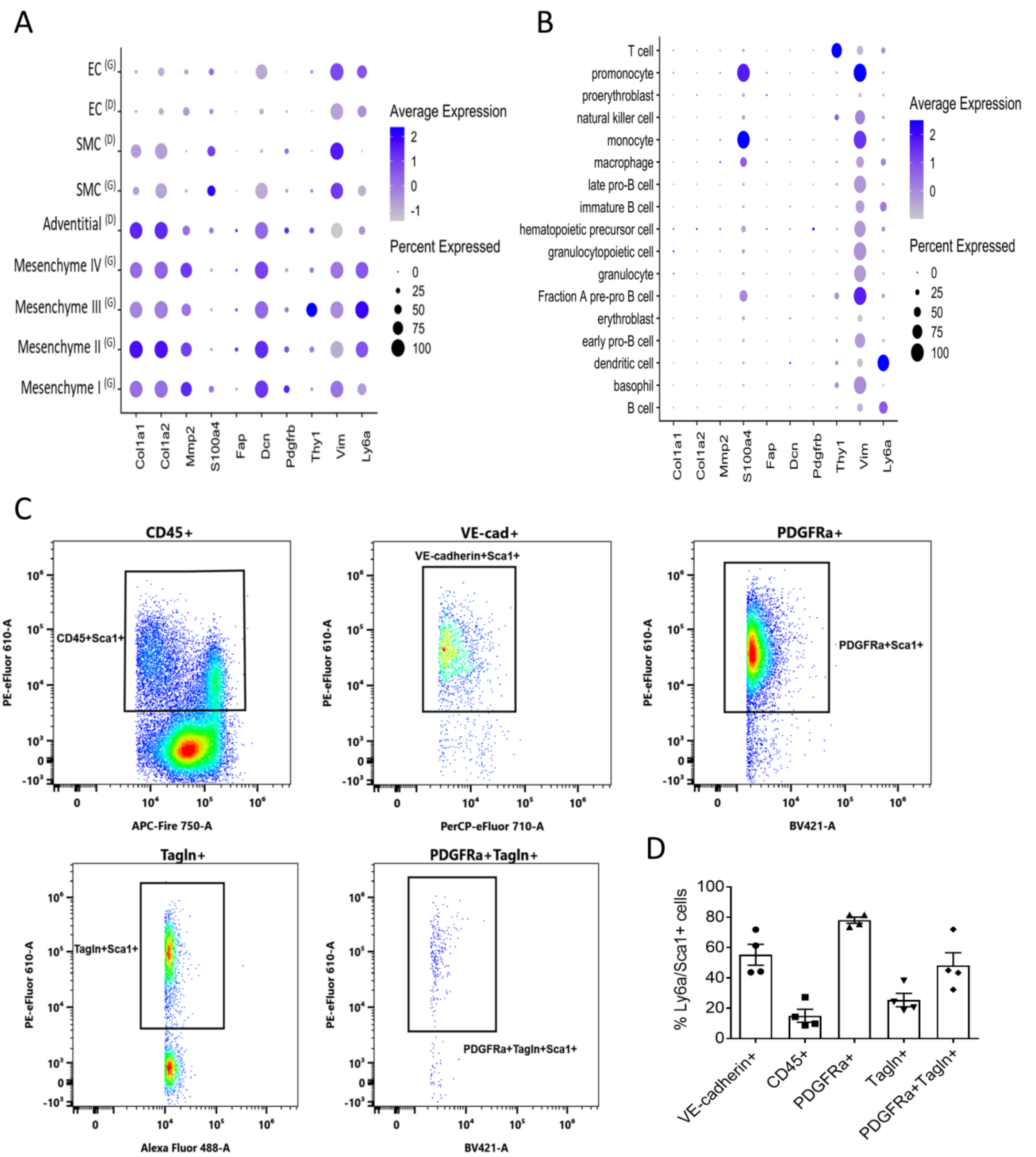

Supplemental figure S2. Expression traditional fibroblast markers not restricted to fibroblasts or mesenchymal cells. A. Expression of traditional fibroblast markers in Gu dataset[56] and Dobnikar dataset[57], **B.** Expression of traditional fibroblast markers in spleen and bone marrow from Tabula Muris[58]. Annotation of cell types is according to the original papers.[56-58] **C-D.** Flow cytometry gating strategy and quantification of Ly6a/Sca-1 positivity in all vascular wall cell types, originating from thoracic aorta adventitia (N=4 groups, 7 young C57Bl6/J mice per group, total 28 mice). All results show mean ± SEM.

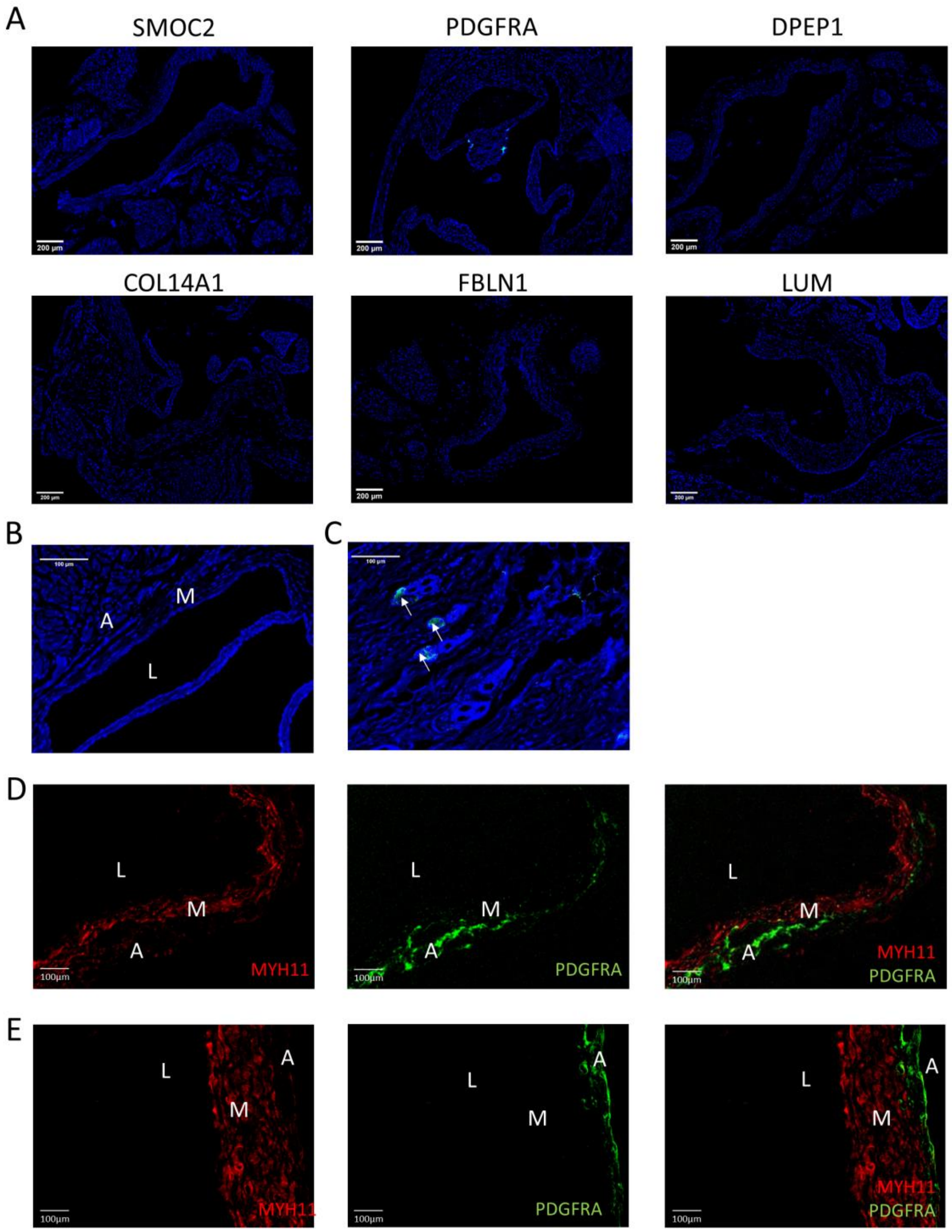

Supplemental Figure S3. Negative controls, immunohistochemical analysis of CCL11 in healthy C57BL/6J aortic roots, PDGFRA in Myh11-reporter mice and MYH11 in Pdgfra-reporter mice. A. Negative controls for immunohistochemical stainings of fibroblast signature markers, **B.** Murine aortic root immunohistochemically stained for Ccl11. Adventitia indicated by A, Media by M and Lumen by L., **C.** Positive control, murine dermis, with Ccl11 expression in green. **D.** PDGFRA expression in myosin heavy chain 11 (MYH11) reporter brachiocephalic artery. **E.** MYH11 expression in aortic root of Pdgfrα-TdTomato reporter. Myh11 in red, Pdgfrα in green, co-colaozaition (yellow) is absent. A indicates adventitia, M indicates media, and L indicates lumen.

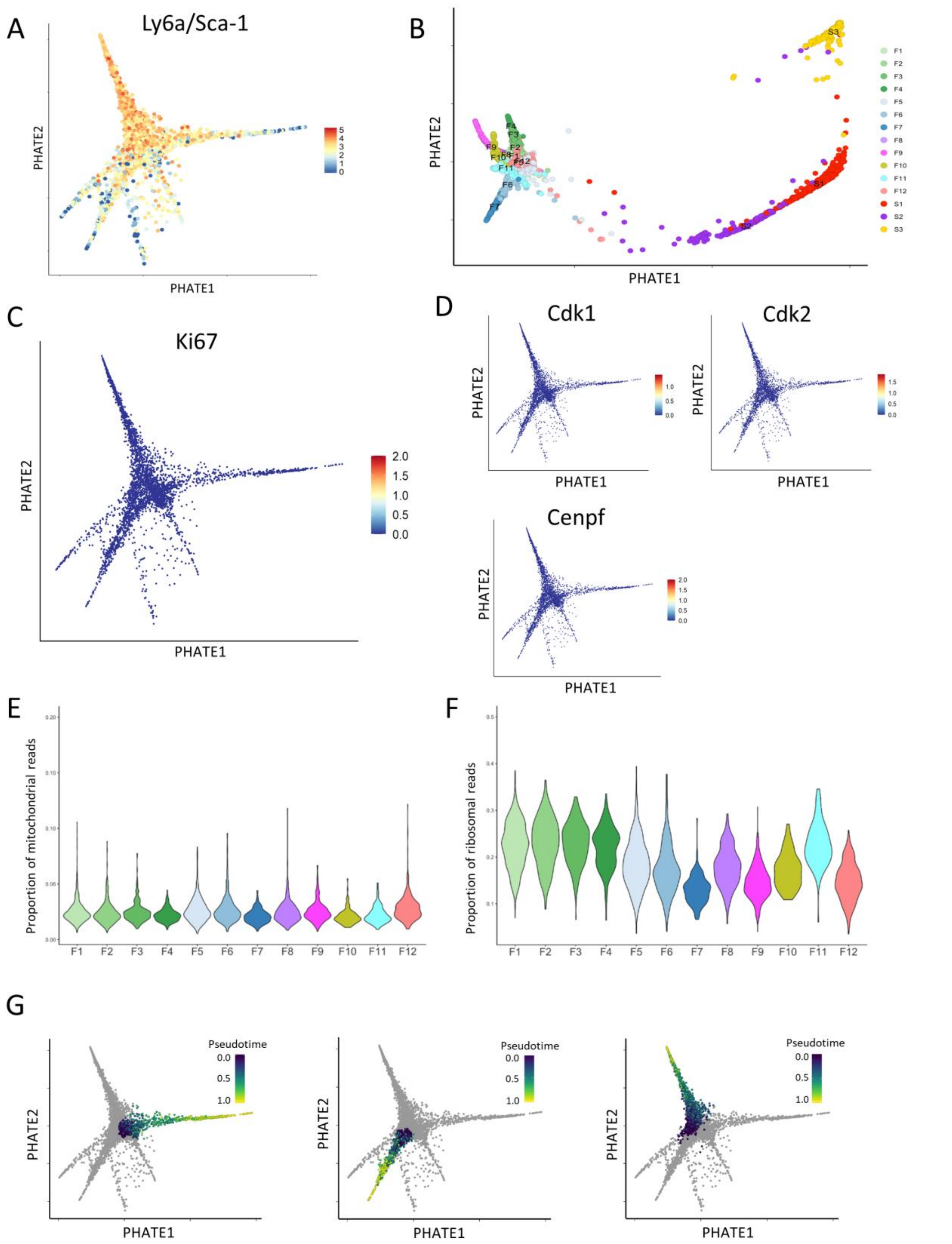

Supplemental figure S4. Expression of proliferation markers, mitochondrial genes or ribosomal genes absent in fibroblasts. A. *Ly6a/Sca-1* expression projected on PHATE plot of Figure 3A, **B.** PHATE dimensionality reduction on total dataset including fibroblasts and SMCs, **C.** *Ki67* expression projected on PHATE plot of Figure 3B, **D.** Expression of proliferation markers *Cdk1*, *Cdk2* and *Cenpf* projected on PHATE plot of Figure 3B, **E.** Proportion of mitochondrial genes among the twelve fibroblast clusters, **F.** Proportion of ribosomal genes among the twelve fibroblast clusters, **G.** Monocle pseudotime projection on each PHATE plot from figure 3B.

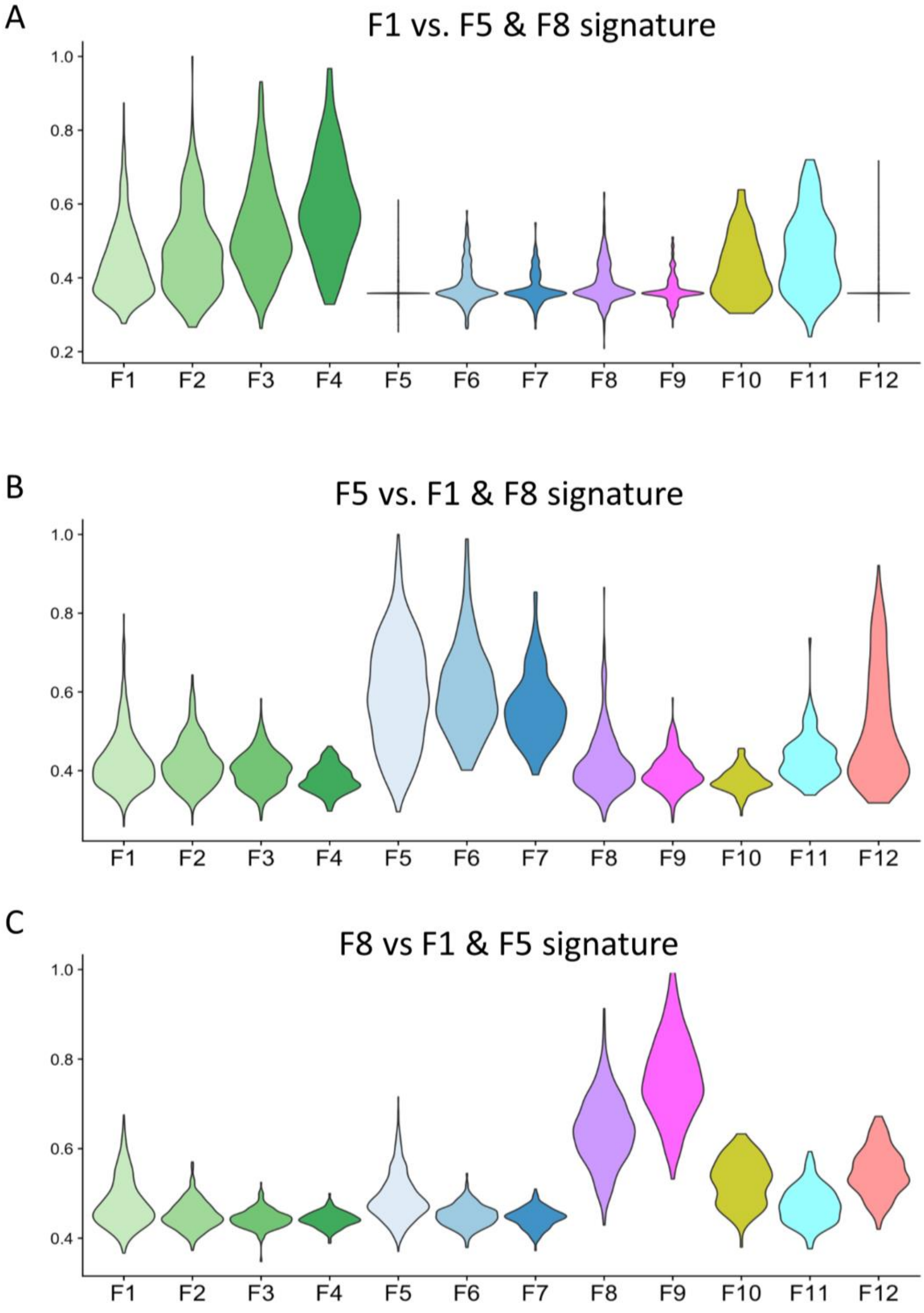

Supplemental figure S5. Gene signatures for the different core clusters per differentiation trajectory. A. Differential expression of signature for F1 vs F5+F8 in clusters F2, F3, F4 , and to a lesser extent F10 and F11, suggest these originate from F1, **B.** Differential expression of F5 vs F1+F8 signature in clusters F6, F7 and F12, **C.** Differential expression of F8 vs F5+F1 signature in population F9

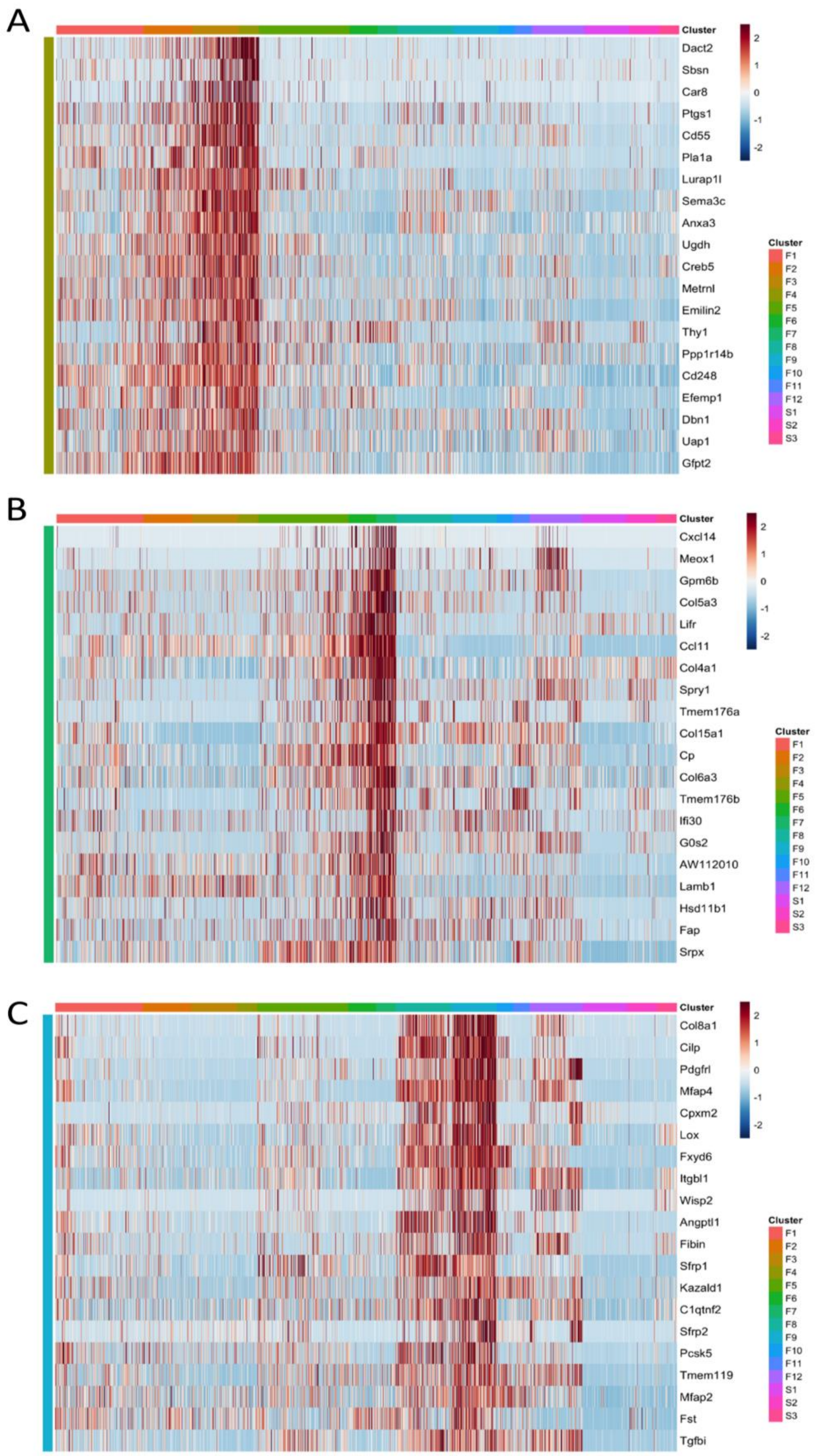

Supplemental Figure 6. Heatmaps depicting gene expression of trajectory specific markers. Heatmap for differentially expressed genes of trajectory 1 in **A**, trajectory 2 in **B** and trajectory 3 in **C**. Criteria included expression of genes in >70% of cells in end-cluster of each trajectory and <35% of remaining cells.

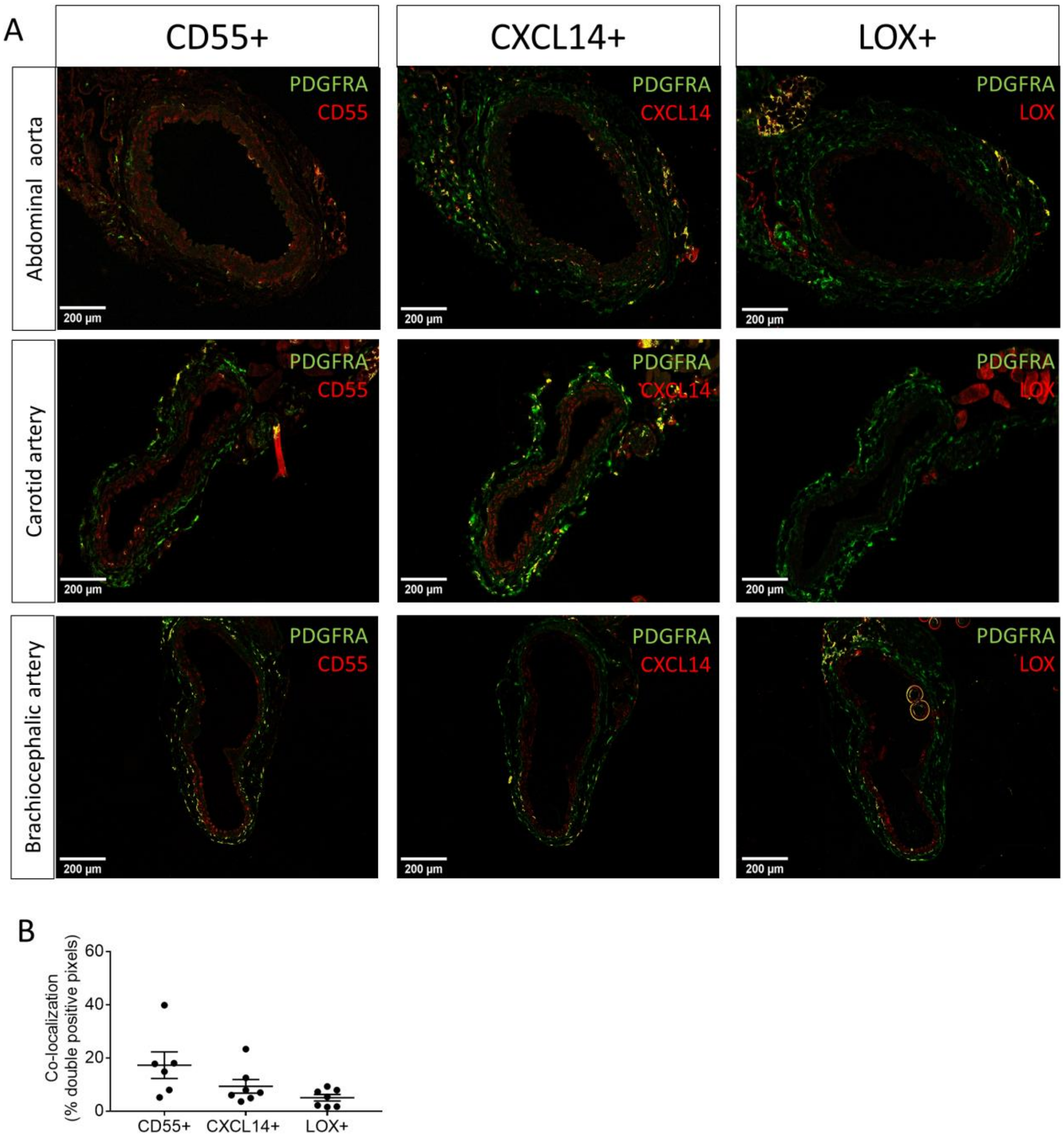

Supplemental Figure 7. Markers representing differentiated clusters presence in multiple vascular beds A. Immunohistochemical analysis of markers representing differentiated clusters for each trajectory in abdominal aorta, carotid artery and brachiocephalic artery (BCA), **B.** Quantification of co-localization of trajectory markers CD55, CXCL14 and LOX with PDGFRA in adventitia of healthy C57BL/6J brachiocephalic arteries. All results show mean ± SEM.

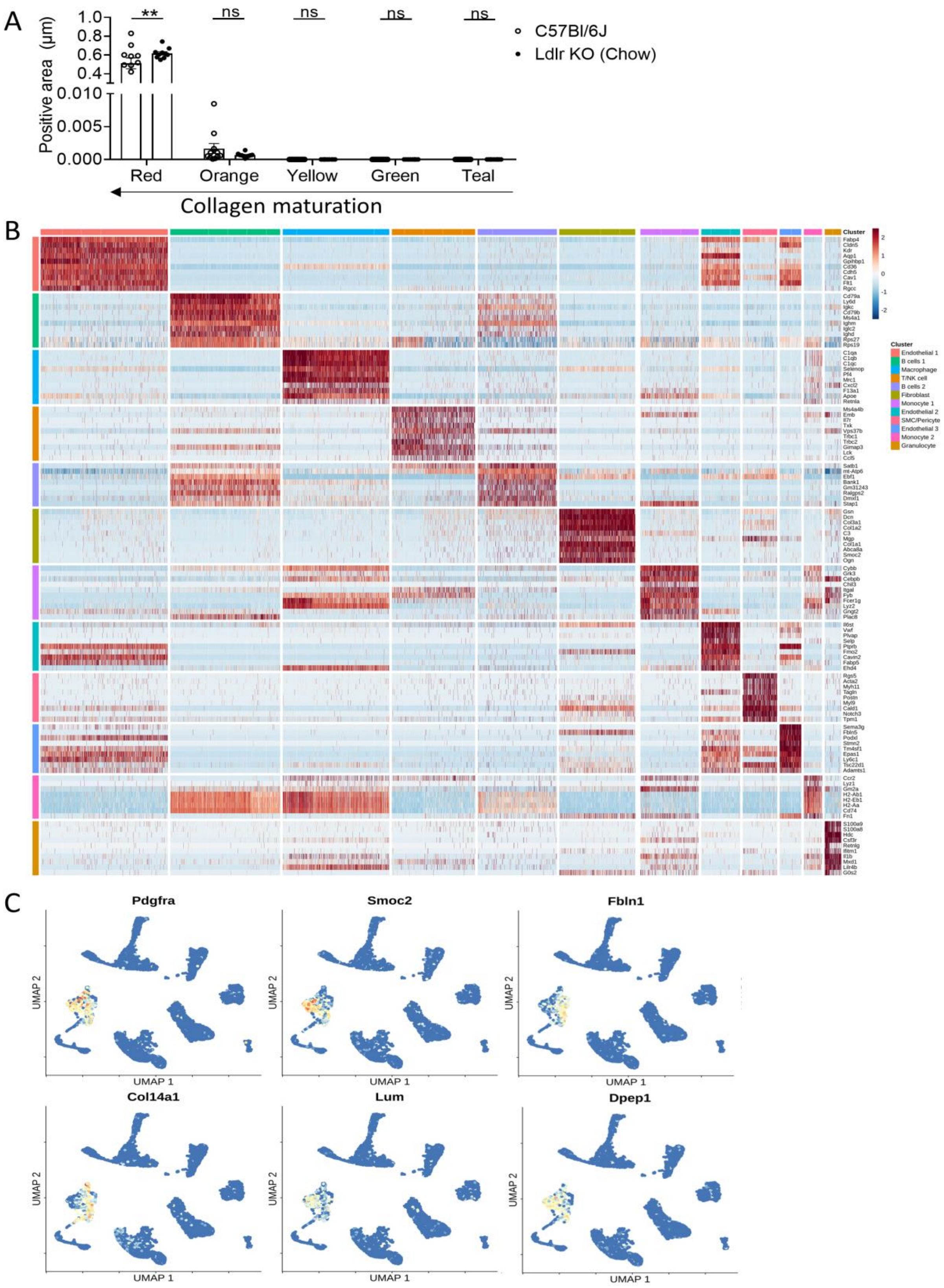

Supplemental Figure S8. Single cell sequencing of adventitia *Ldlr* KO mice A. Sirius red staining in adventitia of healthy C57BL/6J and Ldlr KO BCA. Red represents mature collagen, while teal presents the least mature collagen. **B.** Heatmap for cell annotation of single cell sequencing data, originating from *Ldlr* KO mice on chow and high cholesterol diet for 16 weeks, **B.** Annotation of fibroblasts in *Ldlr* KO single cell sequencing dataset making use of fibroblast-specific markers identified in Figure 2A. Visualization in UMAP. Statistical analyses were performed using two-way ANOVA with Bonferroni post-hoc test (A). All results show mean ± SEM **p<0.015

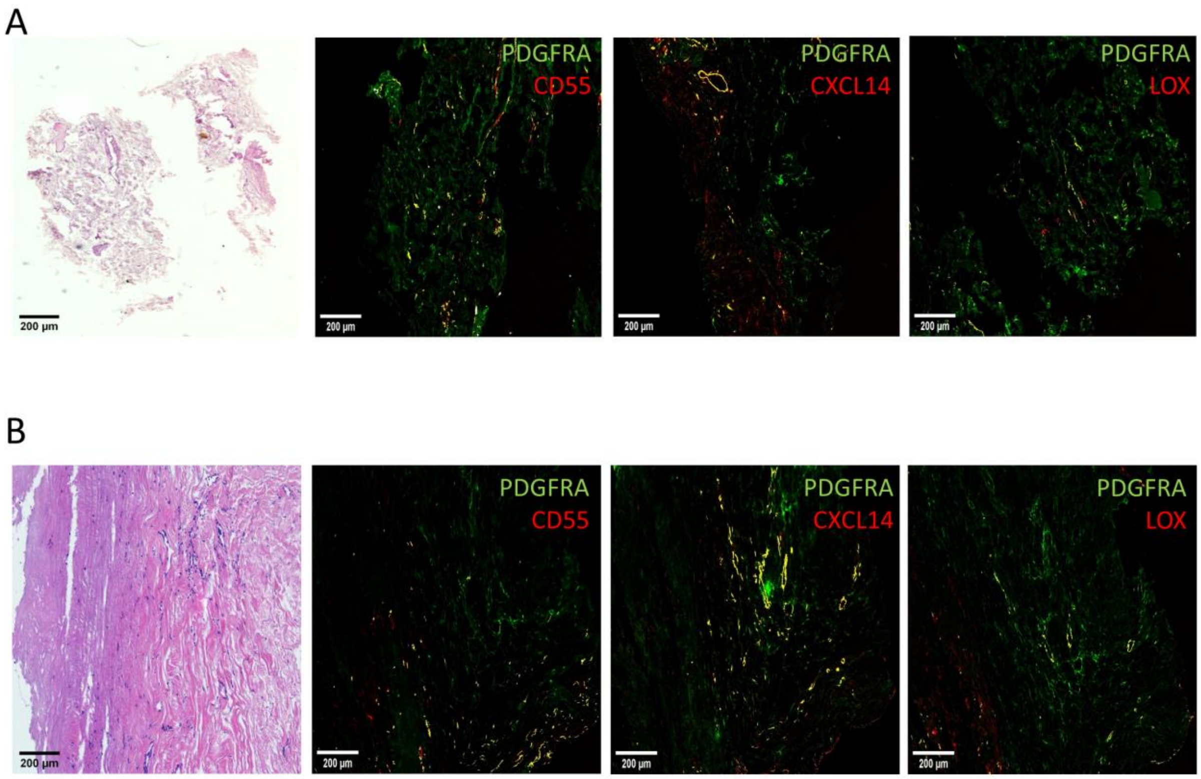

Supplemental Figure 9. Fibroblast trajectories in human specimens.
A. Immunohistochemical stainings of CD55+fibroblasts, CXCL14+fibroblasts, and LOX+ fibroblasts representing trajectory 1-3 respectively in specimens from carotid anastomosis during aortic bypass surgeries, **B.** Trajectory presence in human carotid adventitia, obtained from the opposite side of the culprit plaques during carotid endarterectomy, with corresponding H&E. Overlap between PDGFRA and trajectory markers is shown in yellow.

Legend Supplemental Video S1

Dissection of adventitia of the thoracic aorta (ranging from the aortic root until the diaphragm), which was carefully microscopically dissociated from the underlaying medial layer.

Supplemental Methods

Flow cytometry and cell sorting
Adventitia of the thoracic aorta (ranging from the aortic root until the diaphragm (Supplemental Video S1)) was carefully microscopically dissociated from the underlaying medial layer and collected in ice-cold PBS. Adventitial tissue of C57BL/6J or *Ldlr* KO mice was enzymatically digested for 15 minutes at 37°C using collagenase B (0.00284g/ml, Sigma 110088807001), pronase (0.01g/ml, Sigma 10165921001) and DNAse (0.1mg/ml, Roche 11284932001). This enzymatic cocktail ensures optimal isolation of mesenchymal cells[59]. Tissue was filtered through a 70µM strainer and subjected to red blood cell lysis (8.4g NH_4CL + 0.84g $NaHCO_3$ in 1 liter H_2O, pH 7.2-7.4). Living, DAPI-negative, mesenchymal cells were sorted as CD45 negative (BioLegend, 103114), and ICAM2 negative cells (BioLegend, 400526) on FACS Aria III for scRNA-seq in case of 8 week old C57BL/6J mice or living, DAPI-negative, cells for Ldlr KO mice.

Cells isolated from adventitia originating from either young C57BL/6J mice (8 weeks, male), aged C57BL/6J mice (72 weeks, male), *Ldlr* KO mice on chow or high cholesterol diet for 16 weeks were used for protein validation using flow cytometry (FACS canto II). After FC receptor blocking (15246827, Thermofisher) were stained with the following antibodies: CD45 (Biolegend, 103154), Cdh5/VE-cadherin (Invitrogen, 53-1441-82 or eBioscience 46-1441-82), Transgelin (Novus biologicals, NBP2-47689PCP or NBP2-47689AF488), Platelet derived growth factor alpha (PDGFRA) (BD Pharmingen, 562774), Sca-1/Ly6a (eBioscience 61-5981-82), CD55 (Biolegend, 131804), CXCL14 (Abcam, ab264467) and Lysyl oxidase (LOX) (Novus biologicals, NB-100-2527AF647), live/dead fixable cell stain (Invitrogen, L34957). In case of CXCL14, the antibody was labelled using a PE/Cy7 conjugation kit (Abcam, ab102903). For intracellular stainings (Transgelin, CXCL14 and LOX), fix & perm cell permeabilization kit was used (Invitrogen, GAS004). Data analysis was performed with BD FACS Diva software.

Single-cell sequencing
After cell count number and viability check with trypan blue (>85%), a total of ~16.000 adventitial CD45-/ICAM2- cells from healthy 8 weeks old, male C57BL/6J mice were loaded on a chromium single-cell controller using V2 reagent kit (10X Genomics). In case of *Ldlr* KO, a total of ~15.000 cells were loaded using V2 reagent kit (10X Genomics). Samples were loaded approximately 4 hours after tissue isolation. Libraries of cDNA were synthesized as suggested by 10X genomics and used to create sequencing libraries. In short, in reaction vesicles (gel beads in emulsion, GEMs), cells were lysed and barcoded oligonucleotides reverse transcribed before clean-up and cDNA amplification. The Chromium Single-Cell 3' Library Kit was then used to generate indexed sequencing libraries. Sequencing was performed on Illumina HiSeq4000. In case of C57BL/6J, 5701 cells were yielded with ~87,000 reads per cell and for *Ldlr* KO, 4800 cells were yielded after chow diet and ~8000 cells after HCD, with 63,000 and 47,390 reads per cell respectively (Supplemental Tables 1-2).

Immunohistochemical stainings
Murine tissue was fixed in 1% paraformaldehyde overnight, paraffin-embedded, and serially sectioned (4µm). For stainings, only sections that had mature media (determined by elastin fiber presence) were used. Tissue was deparaffinized using Xylene and rehydrated using an alcohol gradient (100-50% in dH_2O). Antigen retrieval was performed using low pH EnVision Dako target retrieval solution (Dako K800521-2), followed by blocking in 10% normal swine serum (Dako, X0901) in tris buffered saline (TBS). Immunohistochemical detection of the following antigens was performed: SMOC2 (Biorbyt, orb525072), COL14A1 (Novus biologicals, NBP2-15940), mouse PDGFRA (R&D, BAF1062), human PDGFRA (R&D, AF-307-NA), FBLN1 (Human Protein Atlas, HPA001613), LUMICAN (Abcam, ab168348), CCL11 (R&D, AF-420-NA), DPEP1 (Abcam, ab121308), MAC3 (Becton Dickinson), CD55 (ThermoFisher, PA5-78991), mouse CXCL14 (Abcam, ab13741), human CXCL14 (Proteintech, 10468-1-AP), LOX (Novus Biologicals, NB100-2527), Collagen type I (Abcam, ab21286), Vimentin (Abcam ab92547), CD90 (Biolegend 105307), and total collagen

(Picosirius red, Polyscience 09400). Rabbit host primary antibodies were detected with a swine anti-rabbit secondary antibody (Dako, E0431), goat host primary antibodies were detected with a rabbit anti-goat secondary antibody (Dako, E046601-2), followed by signal amplification using Vectastain-ABC (Vector, AK-5000). Visualization was performed with 3,3'-diaminobenzidine (DAB, Agilent K346811-2) for single stains, while double stains were visualized with Vector Red/Blue (Vector, SK5100/5300). Pseudo-fluorescent images were created and adventitial co-localization quantified using the Nuance Multispectral Imaging System or Fiji. Quantification of adventitial area (Defined as the area where medial elastin fibers end and the width is roughly similar to the width of the media), collagen 1 content (% adventitial area), and MAC3 (n/mm2 adventitia) was done on images scanned with the Histotech P1000 scanner and analyzed with Qupath (v0.2.0-m8), while Sirius red was quantified on 20X images using Leica Qwin software. Representative images were selected based on the mean value of the corresponding analysis. Please see the Major Resources Table in the Supplemental Materials for additional details on antibodies.

Human sample analysis
Human tissue collection was part of the Maastricht Pathology Tissue Collection (MPTC) and further storage and use of the tissue was in line with the Dutch Code for Proper Secondary use of Human Tissue and the local Medical Ethical Committee (protocol number 16-4-181). This code (https://www.federa.org/codes-conduct) entails an opt-out arrangement and hence tissues were not used in case of objection. The applicability of this code for this study was approved by the Maastricht University hospital (MUMC) local Medical Ethical Committees. Human studies conducted by Li et al.[60] and Wirka et al.[61] are approved by Institutional Review Board at Baylor College of Medicine and Stanford University Institutional Review Board, respectively, and follow the guidelines of the Declaration of Helsinki. Written informed consent was provided by all participants or the organ donors' legal representatives before enrollment. Formalin-fixed, paraffin-embedded (FFPE) carotid arteries were collected at autopsy (n=10), or from patients undergoing carotid endarterectomy (CEA) (n=63 plaques, 43 patients), opposite the plaque (n=10), or at carotid anastomosis during aorta bypass surgery (n=10). 5mm-segments were alternated with frozen segments for histology and RNA isolation in case of CEA. A total of 43 plaque segments were collected from 23 symptomatic patients undergoing CEA in the Maastricht Human Plaque Study (MaasHPS) were used for further microarray analysis. Library preparation, RNA extraction, data processing, normalization and additional information concerning plaque traits have been described in great detail elsewhere[62,63].

Quantification and Statistical analysis

Single-cell sequencing analysis C57BL/6J mice
The 10X Cell Ranger pipeline (v2.1.1) was used to perform alignment of raw sequencing reads to the mouse reference genome (mm10), filtering, barcode, and unique molecular identifiers (UMI) counting. Generated filtered expression matrices were subsequently used for additional quality control and subsequent analysis using the Seurat (v2.3) R package[64]. Initial quality control was performed by removing low quality cells found to express less than 1500 genes, those with a UMI count greater 15,000, or those with more than 15% of reads aligning to mitochondrial genes (mito%, 654 cells removed in total). Global data normalization was then performed using the Normalize Data method[64], which normalizes gene expression in individual cells based on the total gene expression, followed by multiplying by a factor of 10,000, and transforming the data by $\log_e$. Data was then scaled using the ScaleData method[64] and dimensionality reduction was performed using principal component analysis (PCA). PCA was carried out using the most variable genes in the dataset, identified by the FindVariableGenes method[64] selecting genes with a log variance to mean ratio (VMR) greater than 0.1. The appropriate number of principal components to be used for graph-based clustering and t-distributed stochastic neighbour embedding (tSNE) construction was determined by choosing the principal component (PC) after which the standard deviation of subsequent PCs remained approximately constant. Cluster identification was performed using the FindClusters method[64] using PCA as the chosen method of dimension reduction. Identified clusters were then

visualized on a tSNE plot constructed using the appropriate number of PCs. Clusters found to have a low proportion of cells expressing *Pdgfrb* or containing cells positive epithelial markers (*Krt19*, *Lgals7*, and *Cd82*) were removed from the dataset prior to re-clustering as described above (639 cells in total). Identified clusters were categorized based on their marker gene expression as either being smooth muscle (672 cells positive for *Myh11, Acta2, Tagln, Cnn1*) or fibroblast-like (3736 cells positive for *Col1a1, Col1A2, Ly6a, Mmp2*). Differential gene expression analysis compared smooth muscle cells to fibroblasts cells using the FindAllMarkers command [64]. Only genes expressed in a minimum of 33% of cells in the given cell type, with a minimum $\log_e$ fold change (logFC) in expression of 0.25, and with a difference in the fraction of positive cells between groups of at least 33%. Significantly differentially expressed markers were identified by the Wilcoxon rank sum test as having a Bonferroni adjusted P value <0.05. The top 20 markers based on logFC from each cell type were used for heatmap construction. Cell type markers were similar with mito% <10% and <15%.

Following sub-setting of data to contain only fibroblast-like cells, PHATE dimension reduction[65] was performed using the most variable genes in the fibroblast dataset. Highly variable genes were selected with an average expression (quantified as normalized ln(UMI+1)) between 0.05 and 4 and with a log VMR between 0.075 and 10. Cluster identification within the fibroblast dataset was performed using the FindClusters method [64] with PHATE[65] used as the dimension reduction method. Identified clusters were then visualized on the PHATE plot using the DimPlot command [64]. Markers from each fibroblast cluster were identified using the FindAllMarkers method [64] selecting genes only expressed in at least 25% of cells within the given cluster and with a logFC in expression threshold of at least 0.2. Comparative scRNA-seq datasets were imported directly as filtered count matrices and processed in accordance with the methods from the accompanying publications[18,28,30,56].

Single-cell sequencing analysis *Ldlr* KO mice

Filtered count matrices were generated using the 10X CellRanger V3.0.2 pipeline using the standard GRCh38-3.0.0 genome reference downloaded from 10X genomics (10X Genomics, Pleasanton, USA). The R package scater was used to perform cell filtering quality control on individual datasets [66]. Cells with a UMI count exceeding 3 median absolute deviations (MADs) from the median UMI value were excluded from downstream analysis. Similarly, cells with a total gene count less than 200 genes or with a high proportion of reads originating from mitochondrial genes (>4MADs) were also excluded. Prior to combining the two datasets, data normalisaton was performed using the MultiBatchNormalisation method [67]. Mitochondrial and ribosomal genes were excluded from the 2000 highly variable genes identified using the FindVariableFeatures function and the 'vst' selection method in Seurat V3.2.3 [68]. Following scaling of data, principal component analysis was performed using the previously identified list of highly variable genes. Clustering of cells was performed using the standard 'FindNeighbours' and 'FindClusters' methods including the first 12 principal components [68]. Clustered data was then visualised in two dimensions using the Manifold Approximation and Projection (UMAP) method calculated using the 'RunUMAP' command [68]. Differential gene expression analysis was performed using the 'FindAllMarkers' method selecting markers expressed in at least 30% of cells in the corresponding cluster and with a minimum log fold change in expression of 0.3 compared to the remainder of the dataset. Count data from cells belonging to the identified fibroblast cluster was extracted to further explore fibroblast heterogeneity using the same processing steps described above. Contaminating schwann and mesothelial cells were excluded from further analysis of fibroblast heterogeneity. PHATE reduction analysis was performed as described below [69]. Published datasets were reanalysed per published methods[56, 57, 60, 61, 70, 71].

Cell signature scores

Cell signature scores were calculated as the scaled geometric mean of the expression of selected marker genes within each cell. All gene names within the dataset beginning with 'Mt' were included for generating the mitochondrial signature. All gene names beginning with 'Rpl' or 'Rps' within the dataset were included for calculating the ribosomal signature.

Pseudotime and RNA velocity analysis

Pseudotime cellular trajectories were calculated with the Monocle package (v2.10.1)[72]. Subsets of fibroblast cells were first produced based on the localization of clusters within the branches of the previously generated PHATE plot. The FindMarkers method[64] was then used to identify markers of clusters localizing at the beginning and end of each PHATE branch. Marker genes with the highest logFC in expression were subsequently used for dimensionality reduction of data to two dimensions using the reduceDimension method[72]. Pseudotime values were then calculated using the orderCells command applying default Monocle parameters[72]. Following scaling from 0 to 1, pseudotime values were subsequently mapped onto the corresponding cells on the previously generated PHATE plots. Directionality of cellular transitions were inferred by calculating the RNA velocity of individual cells using the velocyto R package[73]. Reads were identified as mapping to either intronic or exonic sequences using the DropEst pipeline [74] utilising the previously generated binary alignment files from the Cell Ranger pipeline. Velocyto was then used to calculate RNA velocity using KNN pooling with Kcells = 25 and gamma fit performed using the full range of cellular expression magnitudes. RNA velocity vectors were then superimposed onto the previously generated PHATE plot.

Functional analysis using gene ontology (GO) terminology

Functional enrichment analysis was performed using G:profiler[75]. A ranked list of the differentially expressed genes per end cluster was used as input. To increase the interpretative value, the size of the functional category range was set from 5 to 750. Electronic GO annotations were disabled and the size of query/term intersection was set to 3 to increase the reliability [76]. The top-10 Go biological process terms per cluster were selected and plotted on an excel bubble chart where the diameter of the node represents the -log10(p-Value).

Enrichment analysis using hypergeometric testing

The DEGs from the full trajectories (F1, F2, F3, F4, n = 216; F5, F6, F7, n = 235; F8, F9, n = 317) were intersected with 1) GWAS CAD-associated genes, and 2) human aorta fibroblast DEGs from the study of Li et al[60]. For this, a total of 329 CAD-associated genes were retrieved from the GWAS association file (v1.0, 2021-12-07; downloaded from the GWAS Catalog[77] website: https://www.ebi.ac.uk/gwas/) by searching the key word “coronary” in the term “disease/trait”. In addition, for each of the four human aorta fibroblast clusters reported by Li et al., we downloaded the top 20 DEGs from the original paper[60] and combined them as a comprehensive fibroblast gene set. Hypergeometric testing was used to evaluate the statistical significance of the overlap genes between trajectory genes and CAD or fibroblast genes. Mouse genes were converted to human genes by biomaRt R package (v2.50.1)[78].

Data availability

Data are deposited in a repository (GSE196395),and may be inspected on a web-based interface (Plaqview.com)[79]. Count matrices and code are available upon reasonable request.

Statistical analysis

For human samples, correlations between genes and clinical traits were calculated using Pearson’s Correlation Coefficient. Only pairwise complete observations were included if missing values were contained in traits. Student P-value was calculated based on the correlations and sample size. Normality of the data was assessed through D’Agostino-Pearson omnibus normality test and potential outliers were identified through the ROUT method. For mice flow cytometry analysis and Sirius red quantification, an ordinary two-way ANOVA was performed, followed by Tukey’s multiple comparisons test. For immunohistochemistry analyses, depending on number of groups unpaired T-test with Welch’s correction or one-way ANOVA was used, followed by Bonferroni’s multiple comparisons test. Statistical testing was done using Graphpad Prism 7.0.

Supplemental References

56. Gu W, Ni Z, Tan YQ, Deng J, Zhang SJ, Lv ZC, Wang XJ, Chen T, Zhang Z, Hu Y, Jing ZC, Xu Q. Adventitial Cell Atlas of wt (Wild Type) and ApoE (Apolipoprotein E)-Deficient Mice Defined by Single-Cell RNA Sequencing. *Arterioscler Thromb Vasc Biol* 2019;**39**:1055-1071.
57. Dobnikar L, Taylor AL, Chappell J, Oldach P, Harman JL, Oerton E, Dzierzak E, Bennett MR, Spivakov M, Jorgensen HF. Disease-relevant transcriptional signatures identified in individual smooth muscle cells from healthy mouse vessels. *Nat Commun* 2018;**9**:4567.
58. Tabula Muris C. Single-cell transcriptomics of 20 mouse organs creates a Tabula Muris. *Nature* 2018;**562**:367-372.
59. Ramachandran P, Dobie R, Wilson-Kanamori JR, Dora EF, Henderson BEP, Luu NT, Portman JR, Matchett KP, Brice M, Marwick JA, Taylor RS, Efremova M, Vento-Tormo R, Carragher NO, Kendall TJ, Fallowfield JA, Harrison EM, Mole DJ, Wigmore SJ, Newsome PN, Weston CJ, Iredale JP, Tacke F, Pollard JW, Ponting CP, Marioni JC, Teichmann SA, Henderson NC. Resolving the fibrotic niche of human liver cirrhosis at single-cell level. *Nature* 2019;**575**:512-518.
60. Li Y, Ren P, Dawson A, Vasquez HG, Ageedi W, Zhang C, Luo W, Chen R, Li Y, Kim S, Lu HS, Cassis LA, Coselli JS, Daugherty A, Shen YH, LeMaire SA. Single-Cell Transcriptome Analysis Reveals Dynamic Cell Populations and Differential Gene Expression Patterns in Control and Aneurysmal Human Aortic Tissue. *Circulation* 2020;**142**:1374-1388.
61. Wirka RC, Wagh D, Paik DT, Pjanic M, Nguyen T, Miller CL, Kundu R, Nagao M, Coller J, Koyano TK, Fong R, Woo YJ, Liu B, Montgomery SB, Wu JC, Zhu K, Chang R, Alamprese M, Tallquist MD, Kim JB, Quertermous T. Atheroprotective roles of smooth muscle cell phenotypic modulation and the TCF21 disease gene as revealed by single-cell analysis. *Nat Med* 2019;**25**:1280-1289.
62. Jin H, Goossens P, Juhasz P, Eijgelaar W, Manca M, Karel JMH, Smirnov E, Sikkink C, Mees BME, Waring O, van Kuijk K, Fazzi GE, Gijbels MJJ, Kutmon M, Evelo CTA, Hedin U, Daemen M, Sluimer JC, Matic L, Biessen EAL. Integrative multiomics analysis of human atherosclerosis reveals a serum response factor-driven network associated with intraplaque hemorrhage. *Clin Transl Med* 2021;**11**:e458.
63. Jin H, Mees BME, Biessen EAL, Sluimer JC. Transcriptional Sex Dimorphism in Human Atherosclerosis Relates to Plaque Type. *Circ Res* 2021;**129**:1175-1177.
64. Butler A, Hoffman P, Smibert P, Papalexi E, Satija R. Integrating single-cell transcriptomic data across different conditions, technologies, and species. Nat Biotechnol. United States, 2018:411-420.
65. Kevin R. Moon DvD, Zheng Wang, Scott Gigante, Daniel B. Burkhardt, William S. Chen, Kristina Yim, Antonia van den Elzen, Matthew J. Hirn, Ronald R. Coifman, Natalia B. Ivanova, Guy Wolf, Smita Krishnaswamy. PHATE: A Dimensionality Reduction Method for Visualizing Trajectory Structures in High-Dimensional Biological Data. *bioRxiv* 2017.
66. McCarthy DJ, Campbell KR, Lun AT, Wills QF. Scater: pre-processing, quality control, normalization and visualization of single-cell RNA-seq data in R. *Bioinformatics* 2017;**33**:1179-1186.
67. Haghverdi L, Lun ATL, Morgan MD, Marioni JC. Batch effects in single-cell RNA-sequencing data are corrected by matching mutual nearest neighbors. *Nat Biotechnol* 2018;**36**:421-427.
68. Stuart T, Butler A, Hoffman P, Hafemeister C, Papalexi E, Mauck WM, 3rd, Hao Y, Stoeckius M, Smibert P, Satija R. Comprehensive Integration of Single-Cell Data. *Cell* 2019;**177**:1888-1902 e1821.
69. Moon KR, van Dijk D, Wang Z, Gigante S, Burkhardt DB, Chen WS, Yim K, Elzen AVD, Hirn MJ, Coifman RR, Ivanova NB, Wolf G, Krishnaswamy S. Visualizing structure and transitions in high-dimensional biological data. *Nat Biotechnol* 2019;**37**:1482-1492.
70. Cochain C, Vafadarnejad E, Arampatzi P, Pelisek J, Winkels H, Ley K, Wolf D, Saliba AE, Zernecke A. Single-Cell RNA-Seq Reveals the Transcriptional Landscape and

Heterogeneity of Aortic Macrophages in Murine Atherosclerosis. *Circ Res* 2018;**122**:1661-1674.
71. Dawson A, Li Y, Li Y, Ren P, Vasquez HG, Zhang C, Rebello KR, Ageedi W, Azares AR, Mattar AB, Sheppard MB, Lu HS, Coselli JS, Cassis LA, Daugherty A, Shen YH, LeMaire SA. Single-Cell Analysis of Aneurysmal Aortic Tissue in Patients with Marfan Syndrome Reveals Dysfunctional TGF-beta Signaling. *Genes (Basel)* 2021;**13**.
72. Trapnell C, Cacchiarelli D, Grimsby J, Pokharel P, Li S, Morse M, Lennon NJ, Livak KJ, Mikkelsen TS, Rinn JL. The dynamics and regulators of cell fate decisions are revealed by pseudotemporal ordering of single cells. *Nat Biotechnol* 2014;**32**:381-386.
73. La Manno G, Soldatov R, Zeisel A, Braun E, Hochgerner H, Petukhov V, Lidschreiber K, Kastriti ME, Lonnerberg P, Furlan A, Fan J, Borm LE, Liu Z, van Bruggen D, Guo J, He X, Barker R, Sundstrom E, Castelo-Branco G, Cramer P, Adameyko I, Linnarsson S, Kharchenko PV. RNA velocity of single cells. *Nature* 2018;**560**:494-498.
74. Petukhov V, Guo J, Baryawno N, Severe N, Scadden DT, Samsonova MG, Kharchenko PV. dropEst: pipeline for accurate estimation of molecular counts in droplet-based single-cell RNA-seq experiments. *Genome Biol* 2018;**19**:78.
75. Raudvere U, Kolberg L, Kuzmin I, Arak T, Adler P, Peterson H, Vilo J. g:Profiler: a web server for functional enrichment analysis and conversions of gene lists (2019 update). *Nucleic Acids Res* 2019;**47**:W191-W198.
76. Reimand J, Isserlin R, Voisin V, Kucera M, Tannus-Lopes C, Rostamianfar A, Wadi L, Meyer M, Wong J, Xu C, Merico D, Bader GD. Pathway enrichment analysis and visualization of omics data using g:Profiler, GSEA, Cytoscape and EnrichmentMap. *Nat Protoc* 2019;**14**:482-517.
77. Buniello A, MacArthur JAL, Cerezo M, Harris LW, Hayhurst J, Malangone C, McMahon A, Morales J, Mountjoy E, Sollis E, Suveges D, Vrousgou O, Whetzel PL, Amode R, Guillen JA, Riat HS, Trevanion SJ, Hall P, Junkins H, Flicek P, Burdett T, Hindorff LA, Cunningham F, Parkinson H. The NHGRI-EBI GWAS Catalog of published genome-wide association studies, targeted arrays and summary statistics 2019. *Nucleic Acids Res* 2019;**47**:D1005-D1012.
78. Durinck S, Spellman PT, Birney E, Huber W. Mapping identifiers for the integration of genomic datasets with the R/Bioconductor package biomaRt. *Nat Protoc* 2009;**4**:1184-1191.
79. Ma WF, Hodonsky CJ, Turner AW, Wong D, Song Y, Mosquera JV, Ligay AV, Slenders L, Gancayco C, Pan H, Barrientos NB, Mai D, Alencar GF, Owsiany K, Owens GK, Reilly MP, Li M, Pasterkamp G, Mokry M, van der Laan SW, Khomtchouk BB, Miller CL. Enhanced single-cell RNA-seq workflow reveals coronary artery disease cellular cross-talk and candidate drug targets. *Atherosclerosis* 2022;**340**:12-22.
